# Temporal Changes in Sparing and Enhancing Dose Protraction Effects of Ionizing Irradiation for Aortic Damage in Wild-Type Mice

**DOI:** 10.3390/cancers14143319

**Published:** 2022-07-07

**Authors:** Nobuyuki Hamada, Ki-ichiro Kawano, Takaharu Nomura, Kyoji Furukawa, Farina Mohamad Yusoff, Tatsuya Maruhashi, Makoto Maeda, Ayumu Nakashima, Yukihito Higashi

**Affiliations:** 1 Biology and Environmental Chemistry Division, Sustainable System Research Laboratory, Central Research Institute of Electric Power Industry (CRIEPI), Tokyo 201-8511, Japan; nomura@criepi.denken.or.jp; 2 Department of Regenerative Medicine, Division of Radiation Medical Science, Research Institute for Radiation Biology and Medicine, Hiroshima University, Hiroshima 734-8551, Japan; kawano@hiroshima-u.ac.jp (K.-i.K.); drfarinamyusoff@hiroshima-u.ac.jp (F.M.Y.); maru0512@hiroshima-u.ac.jp (T.M.); 3 Biostatistics Center, Kurume University, Fukuoka 830-0011, Japan; furukawa_kyoji@med.kurume-u.ac.jp; 4 Natural Science Center for Basic Research and Development, Hiroshima University, Hiroshima 739-8526, Japan; mmaeda@hiroshima-u.ac.jp; 5 Department of Stem Cell Biology and Medicine, Graduate School of Biomedical and Health Sciences, Hiroshima University, Hiroshima 734-8551, Japan; ayumu@hiroshima-u.ac.jp; 6 Division of Regeneration and Medicine, Medical Center for Translational and Clinical Research, Hiroshima University Hospital, Hiroshima 734-8551, Japan

**Keywords:** ionizing irradiation, aortic damage, fibrosis, intima-media thickening, inflammation, sparing dose protraction effect, enhancing dose protraction effect, temporal change, C57BL6/J

## Abstract

**Simple Summary:**

Ionizing radiation exposure of the circulatory system occurs at various dose rates. Our previous work showed sparing and enhancing effects of dose protraction for aortic changes in wild-type mice at 6 months after starting acute, intermittent, or continuous irradiation with 5 Gy of photons. Here we report that irradiation produces qualitatively similar albeit quantitatively less aortic changes at 12 months than at 6 months after stating irradiation. The magnitude of changes at 12 months was not smaller in 25 fractions (Frs), but was smaller in 100 Frs and chronic exposure, than acute exposure. The magnitude at 6 and 12 months was greater in 25 Frs, smaller in 100 Frs, and much smaller in chronic exposure, compared with acute exposure. These findings suggest that dose protraction changes aortic damage, in a manner that depends on post-irradiation time and is not a simple function of dose rate.

**Abstract:**

In medical and occupational settings, ionizing irradiation of the circulatory system occurs at various dose rates. We previously found sparing and enhancing dose protraction effects for aortic changes in wild-type mice at 6 months after starting irradiation with 5 Gy of photons. Here, we further analyzed changes at 12 months after stating irradiation. Irrespective of irradiation regimens, irradiation little affected left ventricular function, heart weight, and kidney weight. Irradiation caused structural disorganizations and intima-media thickening in the aorta, along with concurrent elevations of markers for proinflammation, macrophage, profibrosis, and fibrosis, and reductions in markers for vascular functionality and cell adhesion in the aortic endothelium. These changes were qualitatively similar but quantitatively less at 12 months than at 6 months. The magnitude of such changes at 12 months was not smaller in 25 fractions (Frs) but was smaller in 100 Frs and chronic exposure than acute exposure. The magnitude at 6 and 12 months was greater in 25 Frs, smaller in 100 Frs, and much smaller in chronic exposure than acute exposure. These findings suggest that dose protraction changes aortic damage, in a fashion that depends on post-irradiation time and is not a simple function of dose rate.

## 1. Introduction

In medical (diagnostic and therapeutic) and occupational settings, ionizing irradiation exposure of the circulatory system occurs at various doses and dose rates. Recent years have seen a renewal of interest in the impact of radiation on diseases of the circulatory system (DCS) as epidemiological evidence is mounting for excess risk of radiogenic DCS at doses and dose rates much lower than previously estimated [1,2,3]. The International Commission on Radiological Protection (ICRP) currently recommends a single nominal dose threshold of 0.5 Gy for DCS regardless of the rate of dose delivery [4], but its dose rate dependence and mechanistic underpinnings are still not clear [5,6]. It is thence critical to address this issue in the radiation oncology, radiology, and radiation protection fields.

We previously examined radiation effects on the circulatory system of wild-type C57BL6/J (B6J) mice within 6 months (mos) post-irradiation with 5 Gy of photons delivered in various regimens [7,8]. Our previous study showed that compared with an acute single exposure delivered in 10 min, prelesional aortic changes at 6 mos after starting irradiation (ASI) were greater when the same dose was given in 25 fractions (Frs) over the span of 6 wks (wks), smaller in 100 Frs over 5 mos, and much smaller in chronic exposure over 5 mos [8]. This suggests that dose protraction changes aortic damage at 6 mos ASI, albeit in a manner that is not a simple function of dose rate [8]. The aim of this study is to further evaluate the dose protraction effect on the circulatory system (inter alia, the aorta) at 12 mos ASI with 5 Gy, and compare temporal changes in biological effectiveness of various irradiation regimens at 6 and 12 mos ASI. The 25 Fr regimen is pertinent to radiotherapy patients who receive (e.g., during thoracic irradiation and re-irradiation for lung cancer [9,10]) ≥ 5 Gy of X-rays conventionally given in 20–30 Frs over 4–6 wks (5 Frs/wk). The 100 Fr regimen is pertinent to tuberculosis (TB) patients treated with lung collapse who received on the order of 100 chest X-ray fluoroscopic examinations over several years (cumulatively up to ~18 Gy) [11]. The chronic exposure regimen is pertinent to nuclear workers whose chronic cumulative dose exceeded 5 Gy [12,13].

We here report that dose protraction confers both sparing and enhancing effects on aortic damage in a fashion depending on timepoints post-irradiation.

## 2. Materials and Methods

### 2.1. Mice, Irradiation, Shipping and Sampling

B6J male mice at 7 wks of age were shipped by car (~48 km, ~1.2 h) from Charles River Laboratories Japan (recently renamed Jackson Laboratory Japan, Kanagawa, Japan) to CRIEPI (Tokyo, Japan), and were acclimated for a week before irradiation.

This study consists of four irradiation regimens, all with 5 Gy (experimental timelines depicted in Figure S1). Just prior to X-ray irradiation, 10 unanesthetized mice at age 8 wks were placed in a 12-compartment pie cage (Natsume Seisakusho, Japan). Mice were exposed to X-rays (260 kVp and 4.5 mA with a 0.5 mm Al and 0.3 mm Cu filter, at a source-surface distance of 52.7 cm), as an acute single dose, or intermittently in 25 daily Frs (0.2 Gy/Fr spread over 42 days) or in 100 daily Frs (0.05 Gy/Fr spread over 152 days) from a Faxitron MultiRad350 irradiator at a dose rate of 0.5 Gy/min. A pie cage was rotated on a turntable during X-ray exposure. Mice were continuously exposed to ^137^Cs γ-rays over 153 days at a dose rate of <1.4 mGy/h (at a source-surface distance of 371 cm) in routine cages. We designate the last regimen “chronic” irradiation because mice received continuous exposure except for a time needed for husbandry (67.6 h in 153 days, 3.1 h/wk). Sham (0 Gy)-irradiated controls were manipulated in parallel with the test (5 Gy irradiated) mice.

At 10.5–11.4 mos ASI, mice were shipped by car and air (~717 km, 6.6 ± 0.6 h) from CRIEPI to Hiroshima University (Hiroshima, Japan), followed by a two wk quarantine period. Both at CRIEPI and Hiroshima University, mice were maintained under a 12 h light/dark cycle (light onset at 8 am) with ad libitum access to food (a normal-fat diet) and water, as described [8].

At age 60 wks (i.e., at 12 mos ASI), mice were weighed, anesthetized with isoflurane, subjected to echocardiography, and perfused transcardially with phosphate-buffered saline (PBS^−^), followed by tissue sampling. Of the collected descending thoracic aorta, the cranial half was evaluated for morphological changes with field-emission scanning electron microscopy (FE-SEM), the caudal half being assessed for cellular and molecular changes with immunofluorescence and histochemical (Masson’s trichrome and Oil Red O) staining.

During the observation period, 10 out of 94 mice died, with a significant decrease in survival only in the “X-rays 5 Gy acute” group (Figure S2). Irradiated mice exhibited a significant decrease in body weight in two regimens (X-rays 5 Gy acute, X-rays in 25 Frs) compared with sham-irradiated mice, and shipping from CRIEPI to Hiroshima caused a decrease in body weight in most groups (Figure S3). At tissue sampling, hearts and kidneys were also weighed (Figure S4). For each mouse, right and left kidneys were weighed together, then their mean was used as kidney weight.

### 2.2. Echocardiography

Anesthetized mice underwent motion/movement-mode (M-mode) echocardiography with a Toshiba Nemio MX SSA-590A ultrasound scanner and a Toshiba PLM-1202S ultrasound probe (Figure S5). Interventricular septal thickness at end diastole (IVSTd), left ventricular dimension at end diastole (LVDd), left ventricular posterior wall thickness at end diastole (PWTd), and left ventricular dimension at end systole (LVDs) were measured as described [8]. Left ventricular ejection fraction (LVEF), left ventricular fractional shortening (LVFS), and left ventricular mass (LVM) were calculated as described [14].

### 2.3. FE-SEM

The cranial half of the descending thoracic aorta was opened longitudinally, fixed and carbon coated, followed by the FE-SEM analysis of the entire area, as described [7,8]. The surface of the normal aortic endothelium exhibited less frequent horizontal waves and more frequent vertical waves (Figure S6A(a)), and the number of crests in such vertical waves was counted in each of the seven fields/mouse (each field corresponds to the entire area of the image taken at 300× magnification, 1 crest/field corresponding to ~7.5 crests/mm^2^). The detached area in the aortic endothelium with sizes in the longer axis of a few tens of microns was designated “detachment”, and that of the order of 100 µm was designated “large detachment”, as described [7,8]. Each mouse was considered positive if one or more such areas existed in the aorta, and such positivity in the group was evaluated independent of the number of such areas in each mouse (this was also the case for rolling leukocytes).

### 2.4. Immunofluorescence and Histochemistry

The caudal half of the descending thoracic aorta was embedded, snap-frozen, and transversally cryosectioned at a 5 µm thickness for staining, and the section was mounted onto a glass slide, as described [7,8].

Dual immunofluorescence was performed for cluster of differentiation 31 (CD31)/platelet endothelial cell adhesion molecule 1 (PECAM-1) stained green and one of the other markers stained red, with cell nuclei counterstained with 4′,6-diamidino-2-phenylindole (DAPI), as described [7,8]. Staining with primary antibodies against CD31, endothelial nitric oxide synthase (eNOS), vascular endothelial cadherin (VE-cadherin), tumor necrosis factor α (TNF-α), CD68, F4/80, CD3, and transforming growth factor β1 (TGF-β1), and secondary antibodies (anti-rabbit and anti-rat) was quantified, the intima-media thickness (IMT) was measured, as described [7,8].

Masson’s trichrome staining was performed where aniline blue stains collagen fibers in the tunica media blue, and measurements of arterial wall area, intensity of aniline blue staining, and IMT were made, as described [7,8]. Oil Red O stains neutral lipids red, thereby visualizing atherosclerotic plaques in the aorta. Oil Red O staining was conducted, with cell nuclei counterstained with Mayer’s hematoxylin (Lillie’s modification), as described [7,8].

### 2.5. Statistical Analysis

Statistical analyses were performed using R statistical software (version 3.6.1, R Foundation, https://www.r-project.org/ accessed on 17 May 2022), where a *p*-value (after Bonferroni corrections for pairwise comparisons using the *t*-test) of < 0.05 was considered significant (*p* < 0.001 presented as **, 0.001 ≤ *p* < 0.05 as *), 0.05 ≤ *p* < 0.1 as marginally significant (presented as #) and *p* ≥ 0.1 as nonsignificant (presented as ns). Black asterisks (*) or pound (#) signs were used for intra-regimen comparisons (e.g., irradiated vs. sham-irradiated groups in each irradiation regimen), whereas blue asterisks or pound signs were used for inter-regimen comparisons of such intra-regimen differences (i.e., the degree of differences in irradiated and sham-irradiated groups between the two regimens) or for intra-regimen differences but at the two timepoints (i.e., 6 vs 12 mos ASI). *p*-values determined by the one-way analysis of variance (ANOVA) using the F-test of homogeneity among the group means, a log-rank test, a one-sample *t*-test, a two-sample (Welch’s) *t*-test for the null hypothesis of equal means, a chi-square test, Fisher’s exact test, Wald test (logistic regression), and Kolmogorov–Smirnov goodness-of-fit test are presented as *p*. The statistical test used is described in figure legends or table footnotes. Each datapoint was obtained from 8–10 mice and is presented as means and standard deviations, unless otherwise stated.

## 3. Results

In the present study, there are 4 irradiation regimens (acute X-rays, X-rays in 25 Frs, X-rays in 100 Frs, and chronic γ-rays) and 33 prelesional endpoints comprised of 20 endpoints for the aorta (4 endpoints for FE-SEM, 12 endpoints for immunofluorescence, and 4 endpoints for Masson’s trichrome staining), 5 endpoints for body or organ weight, and 8 endpoints for echocardiography. The first subsection (Section 3.1) explains the results in 8 groups of B6J mice at 12 mos ASI, with statistical comparisons in each endpoint. The second subsection (Section 3.2) explains the results of the integrative analysis to compare biological effectiveness of four irradiation regimens for prelesional aortic changes in 16 groups of B6J mice at two timepoints (8 groups each at 6 and 12 mos ASI), with statistical comparisons in multiple endpoints. An outline of statistical comparisons is given in each figure legend and is not repeated here.

### 3.1. Responses at 12 Months after Starting Irradiation

**Figure 1:**
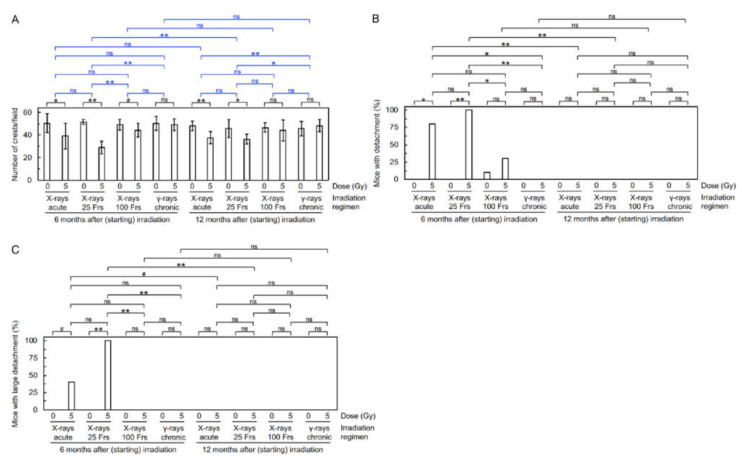
Morphological alterations in the aortic endothelium. Quantitative analysis for (**A**) the number of crests/field, (**B**) percentage of mice with detachment, and (**C**) percentage of mice with large detachment (8–10 mice/group analyzed, Welch’s *t*-test for **A**, Fisher’s exact test for **B**,**C**). Frs, fractions. **, *p* < 0.001. *, 0.001 ≤ *p* < 0.05. #, 0.05 ≤ *p* < 0.1 (marginally significant). ns, *p* ≥ 0.1 (nonsignificant). See footnote in Supplementary Materials for an outline of statistical comparisons. The data in Figure 1A–C for 8 groups at 6 mos ASI were taken from the 2021 Cancers paper [8].

**Figure 2:**
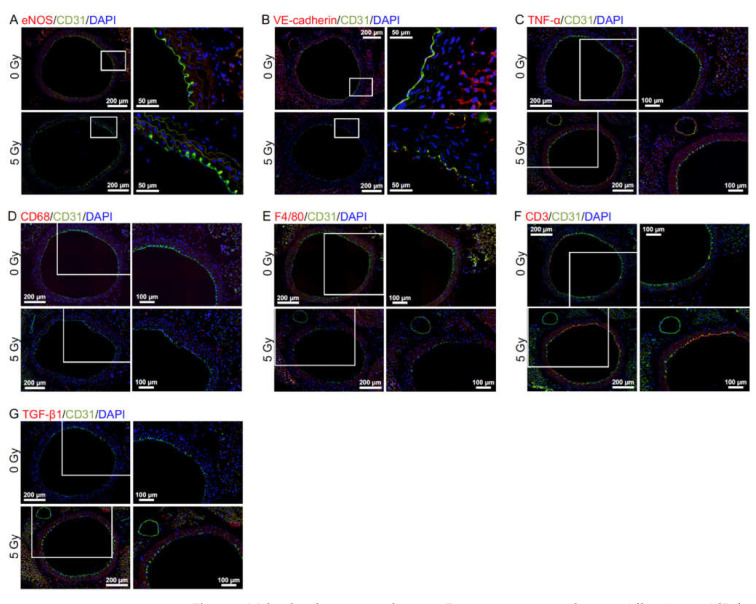
Molecular alterations in the aorta. Representative merged images (all at 12 mos ASI) for double immunofluorescence of CD31 with (**A**) eNOS, (**B**) VE-cadherin, (**C**) TNF-α, (**D**) CD68, (**E**) F4/80, (**F**) CD3 or (**G**) TGF-β1, with cell nuclei counterstained with DAPI. (**A**,**C**–**G**) X-rays in 25 Frs. (**B**) Acute X-rays. Upper panels, 0 Gy. Lower panels, 5 Gy. Boxed areas in the left panels (tiled images) are presented at higher magnification in the right panels. Scale bars are as indicated.

**Figure 3:**
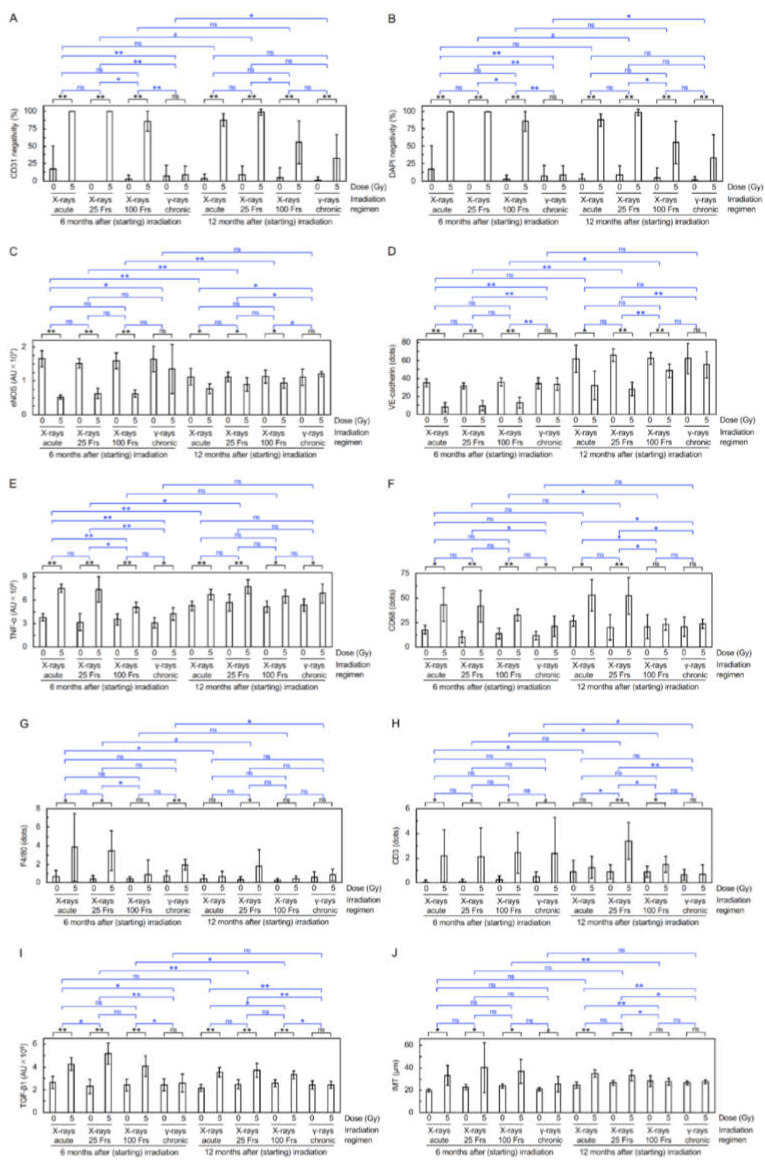
Molecular alterations in the aorta. Quantitative analysis of immunofluorescence for (**A**) CD31 negativity, (**B**) DAPI negativity, (**C**) eNOS, (**D**) VE-cadherin, (**E**) TNF-α, (**F**) CD68, (**G**) F4/80, (**H**) CD3, (**I**) TGF-β1, and (**J**) IMT (8–10 mice/group analyzed, Welch’s *t*-test or Wald test). **, *p* < 0.001. *, 0.001 ≤ *p* < 0.05. #, 0.05 ≤ *p* < 0.1 (marginally significant). ns, *p* ≥ 0.1 (nonsignificant). See footnote in Supplementary Materials for an outline of statistical comparisons. AU, arbitrary unit. Frs, fractions. The data in Figure 3A–J for 8 groups at 6 mos ASI were taken from the 2021 Cancers paper [8]. Representative images are presented in Figure 2.

**Figure 4:**
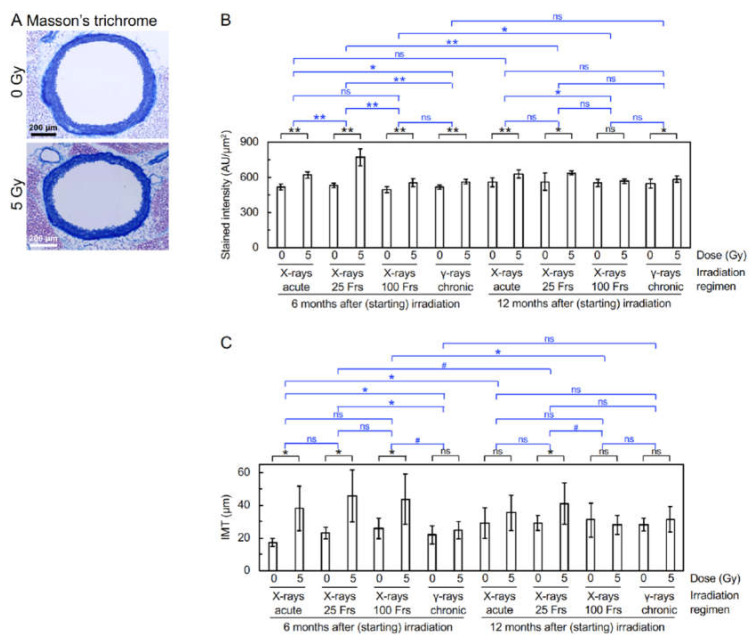
Fibrotic alterations in the aorta. (**A**). Representative images for Masson’s trichrome staining (at 12 mos ASI with 0 Gy or 5 Gy of X-rays in 25 Frs). Scale bars are as indicated. Quantitative analysis for (**B**) intensity of aniline blue (9–10 mice/group analyzed, Welch’s *t*-test). AU, arbitrary unit. Frs, fractions. **, *p* < 0.001. *, 0.001 ≤ *p* < 0.05. #, 0.05 ≤ *p* < 0.1 (marginally significant). ns, *p* ≥ 0.1 (nonsignificant). See footnote in Supplementary Materials for an outline of statistical comparisons. The data in Figure 4 (**B**,**C**) for 8 groups at 6 mos ASI were taken from the 2021 Cancers paper [8].

Compared with sham-irradiated mice, irradiated mice exhibited differences in 27 of 33 endpoints (20 increased, 7 decreased) in ≥ 1 (of 4) irradiation regimens at 12 mos ASI (Table S1), which changed from 24 of 33 endpoints (16 increased, 8 decreased) at 6 mos ASI [8]. Among 13 endpoints for body or organ weight and echocardiography, slight differences were observed in 10 endpoints (6 increased, 4 decreased) in 1–3 (1–2 except for LVEF and LVFS) irradiation regimens at 12 mos ASI (Figures S4A–E and S5A–H), which changed from 5 endpoints (all decreased) in 1 or 3 (1 except for body weight) irradiation regimens at 6 mos ASI [8]: 8 endpoints (heart weight/body weight, kidney weight, kidney weight/body weight, IVSTd, LVDd, LVEF, LVFS, and LVM/body weight) accounted for such differences at 6 and 12 mos ASI. Of 20 endpoints for prelesional aortic changes, differences were found in 17 endpoints (14 increased, 3 decreased) in 1–4 (3–4 in 9 endpoints) irradiation regimens at 12 mos ASI (Figure 1, Figure 2, Figure 3 and Figure 4 and Figures S6–S8), which changed from 18 endpoints (15 increased, 3 decreased) in 2–4 (3–4 in 15 endpoints) irradiation regimens at 6 mos ASI [8]: 3 endpoints (mice with detachment, mice with large detachment, and mice with leukocyte rolling) exhibited such differences at 6 and 12 mos ASI. The next two paragraphs in this subsection focus on such prelesional aortic changes.

FE-SEM analysis was first performed to examine the effect on the morphology of the aortic endothelium. The surface of the aorta in sham-irradiated mice exhibited a morphology of undulations with regular repeating (Figure S6A(a)) that was disturbed in irradiated mice (e.g., manifested as decreased waviness, which was considered due to various accompanying morphological alterations, such as flattening, derangement, and cobblestone formation (Figure S6A(b–d)). Two irradiation regimens (acute X-rays, and X-rays in 25 Frs) reduced waviness at 12 mos ASI, but to a lesser extent than at 6 mos ASI (Figure 1A). Irradiation did not induce detachment and large detachment in the surface of the aorta at 12 mos ASI, in contrast to the case at 6 mos ASI (Figure 1B,C). These findings indicate a repair of radiogenic morphological aortic damage with post-irradiation time.

Immunofluorescent staining was next carried out to evaluate molecular alterations (Figure 2A–G). Regarding responses of vascular endothelial cells (VECs) at 12 mos ASI (Figure 3A–D and Figure S7A–C(a)), 3 or 4 irradiation regimens decreased eNOS (a marker for vascular functionality) and VE-cadherin (a marker for adherens junctions), and increased CD31 negativity (indicative of VEC or CD31 loss), DAPI negativity (indicative of VEC loss), and subcellular fragments (indicating apoptosis). This suggests that irradiation causes vascular damage via partial loss of aortic endothelium of which mechanisms involve apoptosis of VECs. Following 1–4 irradiation regimens, vascular smooth muscle cells (VSMCs) had elevated levels of TNF-α (a marker for proinflammation), CD68 and F4/80 (markers for macrophage), CD3 (a marker for T-cells), and subcellular fragments (indicating apoptosis) (Figure 3E–H and Figure S7A,B,C(b)). This, together with rolling leukocytes observed (Figure S6A(e),B), suggests that radiogenic vascular damage causes inflammation. TNF-α and other factors including ADAM10 have been reported to mediate the degradation and internalization of VE-cadherin, which in turn increases vascular permeability to macromolecules [15,16,17]. Following 2 or 3 irradiation regimens, the aortic wall showed elevated levels of TGF-β1 (a marker for profibrosis), aniline blue stain (a marker for collagen fiber), and IMT (without difference in IMT quantified from images of immunofluorescence or Masson’s trichrome staining) (Figure 3I,J, Figure 4A–C, Figures S8A–C): this suggests that irradiation facilitates fibrosis. Collectively, the present findings suggest that irradiation causes vascular damage and dysfunction, inflammation, and fibrosis (these alterations have all been implicated in the early stages of atherosclerosis). There were smaller differences between irradiated and sham-irradiated groups for these endpoints at 12 mos ASI than at 6 mos ASI.

### 3.2. Temporal Changes in Biological Effectiveness of Four Irradiation Regimens for Prelesional Aortic Changes

This subsection reports on the result of the integrative analysis to compare biological effectiveness of four irradiation regimens (acute X-rays, X-rays in 25 Frs, X-rays in 100 Frs, and chronic γ-rays) at each timepoint (i.e., at 6 and 12 mos ASI), and then to compare biological effectiveness over two timepoints.

#### 3.2.1. Biological Effectiveness at 6 Months after Starting Irradiation

We previously reported that among 18 (out of 20) endpoints for prelesional aortic changes that exhibited differences between irradiated and sham-irradiated mice in ≥ 1 (out of 4 or 5 depending on endpoints) irradiation regimens at 6 mos ASI, 16 endpoints showed differences in ≥ 1 (out of 6 or 10) pairs among irradiation regiments [8]. The integrative analysis of such 16 endpoints suggested biological effectiveness in descending order of X-rays in 25 Frs > acute X-rays > acute γ-rays > X-rays in 100 Frs >> chronic γ-rays [8].

Here we reperformed the integrative analysis for four irradiation regimens (acute X-rays, X-rays in 25 Frs, X-rays in 100 Frs, and chronic γ-rays: i.e., with the data for acute γ-rays excluded), to be consistent with the datasets at 12 mos ASI. Of 18 endpoints that exhibited differences between irradiated and sham-irradiated mice in ≥1 (out of 4) irradiation regimens, 15 endpoints (only IMT-IF excluded from the previous analysis) showed differences in ≥1 (out of 6) pairs among irradiation regiments (Table S2). For these 15 endpoints, we employed two integrative approaches to comparing biological effectiveness of four irradiation regimens. Four different levels of difference (presented as ns, #, *, and ** in the graphs for each endpoint) were changed to discrete scores (0, ±0.5, ±1, and ±1.5) in the first approach (Table S2), suggestive of effectiveness in a descending order of X-rays in 25 Frs > acute X-rays > X-rays in 100 Frs >> chronic γ-rays. Kolmogorov–Smirnov goodness-of-fit test was carried out in the second approach (Table S3), similarly suggesting effectiveness in a descending order of X-rays in 25 Frs > acute X-rays >> X-rays in 100 Frs >> chronic γ-rays. So, the reperformed analysis did not much change the results of the previous analysis where both of the two approaches indicated effectiveness in a descending order of X-rays in 25 Frs > acute X-rays > X-rays in 100 Frs >> chronic γ-rays [8].

#### 3.2.2. Biological Effectiveness at 12 Months after Starting Irradiation

Among 17 (out of 20) endpoints for prelesional aortic changes that exhibited differences between irradiated and sham-irradiated mice in ≥ 1 (out of 4) irradiation regimens at 12 mos ASI, 13 endpoints exhibited differences in ≥1 (out of 6) pairs among irradiation regiments (Table S2). Both of the two integrative approaches for these 13 endpoints suggested effectiveness in a descending order of X-rays in 25 Frs ≥ acute X-rays >> X-rays in 100 Frs ≥ chronic γ-rays (Tables S2 and S4).

#### 3.2.3. Biological Effectiveness at 6 and 12 Months after Starting Irradiation

As aforementioned, 15 endpoints at 6 mos ASI and 13 endpoints at 12 mos ASI showed differences in ≥ 1 (out of 6) pairs among irradiation regiments (Table S2). The first integrative (scoring) approach for these 28 endpoints over two timepoints suggested effectiveness in a descending order of X-rays in X-rays in 25 Frs > acute X-rays > X-rays in 100 Frs > chronic γ-rays (Table S2). The second (Kolmogorov–Smirnov goodness-of-fit test) approach similarly suggested effectiveness in a descending order of X-rays in X-rays in 25 Frs > acute X-rays >> X-rays in 100 Frs > chronic γ-rays (Table S5).

## 4. Discussion

Here we have reported the impacts of irradiation on the circulatory system at 12 mos ASI, for various endpoints, i.e., the weight of the heart and kidneys (Figure S4), echocardiographic alterations (Figure S5), morphological and molecular aortic alterations (Figure 1, Figure 2, Figure 3, Figure 4 and Figures S6–S8). This section focuses on aortic changes, consisting of the following four subsections: the first subsection on difference over two timepoints, the second subsection on inter-regimen differences, the third subsection on lesional aortic changes, and the last subsection on age-dependent aortic changes in non-irradiated or sham-irradiated mice.

### 4.1. Difference at 6 and 12 Months after Starting Irradiation

The frequency of mice with electron microscopically observed detachment and large detachment in the aortic surface increased with time up to 6 mos ASI [7,8], and returned to a baseline level at 12 mos ASI (Figure 1B,C), demonstrating the temporal kinetics for production and a repair of such radiation-induced morphological damage to aortic endothelium. On the other hand, the level of fluorescent microscopically observed partial loss of aortic endothelium (manifested as elevations in CD31 negativity, DAPI negativity, and VEC apoptosis, as well as reductions in eNOS and VE-cadherin) became high within 1 mo ASI, and remained unchanged up to 6 mos ASI [7,8]: then, the level at 12 mos ASI was lower than that at 6 mos ASI but was still higher than a baseline level, particularly in the case of the “acute X-rays” and “X-rays in 25 Frs” regimens (Figure 3A–D and Figure S7A–C(a)), demonstrating a temporal difference between detachment and loss of aortic endothelium. It remains unclear whether these phenomena observed with different approaches are the same with some mechanistic overlaps, but persistent signaling stemming from residual damage may lead to elevations of inflammation, macrophages, and fibrosis (Figure 3E–J and Figure S7A–C(b), Figure 4A–C, Figures S6A(e),B, S7A–C(b) and S8A–C). Such persistent signaling and age-dependent changes may account, at least in part, for the smaller difference between irradiated and sham-irradiated groups at 12 mos ASI than at 6 mos ASI.

### 4.2. Inter-Regimen Difference in Prelesional Aortic Changes

The integrative approaches predicated on various (13–28) aortic endpoints suggest that dose protraction changes aortic damage, albeit in a fashion that depends on post-irradiation time and is not a simple function of dose rate. At 6 mos ASI, the analysis suggested effectiveness in a descending order of X-rays in 25 Frs > acute X-rays > (or >>) X-rays in 100 Frs >> chronic γ-rays (Tables S2 and S3). At 12 mos ASI, the analysis suggested effectiveness in a descending order of X-rays in 25 Frs ≥ acute X-rays >> X-rays in 100 Frs ≥ chronic γ-rays (Tables S2 and S4). Such a slight difference at two timepoints may be attributable, at least in part, to the generally smaller magnitude of radiogenic changes in the aorta at 12 mos than at 6 mos ASI (Figure 1, Figure 3, Figure 4, and Figures S6–S8). The discussion below focuses on the pattern of dose protraction effects when changes over 6 and 12 mos ASI were considered together, namely, effectiveness in a descending order of X-rays in X-rays in 25 Frs > acute X-rays > (or >>) X-rays in 100 Frs > chronic γ-rays (Tables S2 and S5). Of note, the present analysis with Kolmogorov–Smirnov goodness-of-fit test (Tables S3–S5) considered only endpoints with an inter-regimen difference (15 endpoints at 6 mos, 13 endpoints at 12 mos, i.e., 28 endpoints over 6 and 12 mos, as listed in Table S2) in accordance with the previous approaches [8], but the further analysis with Kolmogorov–Smirnov goodness-of-fit test, taking account of all endpoints (20 endpoints each at 6 and 12 mos, i.e., 40 endpoints over 6 and 12 mos, as listed in Table S6), yielded similar patterns of dose protraction effects each at 6 and 12 mos (Tables S7 and S8) and the same pattern over 6 and 12 mos (Table S9).

#### 4.2.1. Enhancing (Inverse) Dose Protraction Effect after X-Rays in 25 Fractions vs. Acute X-Rays

Tables S2 and S5 suggest that the “X-rays in 25 Frs” regimen is more effective compared with the “acute X-rays” regimen, indicating enhancing dose protraction effects for vascular damage in vivo. There is supportive in vivo evidence for higher effectiveness of exposures to 5 Gy of γ-rays in 25 Frs than acute γ-rays for induction of mitochondrial DNA common deletion, senescence-associated β-galactosidase, and p21 expression in kidneys of B6J mice [18]. Reportedly, irradiation of human umbilical vein endothelial cells in vitro with 0.5 Gy of X-rays in two Frs also increased production of reactive oxygen species, phosphorylation of p65 (indicating nuclear factor κB activation), induction of intercellular adhesion molecule 1, and adhesion to polymorphonuclear leukocytes, in comparison with acute X-rays [19]. Such biological observations are further supported by epidemiological findings. For example, the excess relative risk per unit absorbed dose (ERR/Gy) estimates for ischemic heart disease (IHD) mortality in the Canadian TB fluoroscopy cohort of patients who received highly fractionated X-ray exposures, were elevated with a decrease in dose rate when a lag of 10 years was employed, e.g., ERR/Gy (95% confidence limits) of 0.01 (–0.043, 0.078) at 0.3–7.3 Gy/year, 0.145 (0.007, 0.32) at 0.15–0.29 Gy/year, and 0.592 (0.004, 1.4) at 0.0004–0.14 Gy/year [20]. Moreover, a meta-analysis for radiogenic IHD and cerebrovascular disease has indicated a greater risk per unit dose for exposures in more fractions and at a lower dose rate [1]. Furthermore, a reduction in the dose from 1 Gy to 0.01 Gy nearly doubled lifetime excess risk per unit dose estimated from DCS mortality data in the acutely exposed Japanese atomic bomb survivors [21]: this, together with a somewhat similar in vivo evidence of cardiovascular disease mortality after ^60^Co γ-ray exposure [22], indicates enhancing dose protraction effect.

#### 4.2.2. Sparing Dose Protraction Effect after X-Rays in 100 Fractions and Chronic γ-Rays Vis-à-Vis Acute X-Rays

Tables S2 and S5 suggest that the “X-rays in 100 Frs” regimen is less effective compared with the “acute X-rays” regimen and is much less effective compared with the “X-rays in 25 Frs” regimen. Such sparing effects follow long-held dogma in radiation biology that biological effectiveness of photons and other types of low-linear energy transfer (LET) radiation decreases as a dose rate decreases [23]. In addition, Tables S2 and S5 suggest that the “chronic γ-rays” regimen is less effective compared with the “X-rays in 100 Frs” regimen, in spite of the same cumulative dose given over the same span (i.e., 153 days) for these two regiments, whereas one cannot exclude the possibility that radiation quality effect plays a role. Future studies should address if any “boundary” dose protraction regimen exists below which sparing effects are seen, above which enhancing protraction effects are seen (e.g., somewhere between 25 and 100 Frs as suggested here), so does any “boundary” dose protraction regimen that gives no difference in effects with chronic exposure (e.g., > 100 Frs as suggested here).

#### 4.2.3. Limitations

Besides inter-group, inter-regimen, and temporal differences as abovementioned, there are several limitations in our experimental design that need to be further improved. First of all, inconsistent radiation sources were used among irradiation regimens, e.g., with no acute exposure and fractionated exposures to ^137^Cs γ-rays due partially to inter-institutional differences in infrastructures (i.e., a low dose rate irradiator available at CRIEPI vis-à-vis a high dose rate irradiator available at Hiroshima University); we did not carry out chronic X-ray exposure either; however, it is technically not feasible. Secondly, the irradiation span in the “X-rays in 25 Frs” regimen (i.e., 42 days) was not consistent with the “X-rays in 100 Frs” and “chronic γ-rays” regimens (i.e., 153 days). Thirdly, age at acute X-ray exposure did not include 14 wks (upon completion of irradiation with X-rays in 25 Frs) and 30 wks (upon completion of irradiation with X-rays in 100 Frs and chronic γ-rays). Last, several dose points (e.g., a low dose of 0.05 Gy and a moderate dose of 0.5 Gy besides 5 Gy) were not included in the dose response.

The entire effects not merely from the aorta but even from diverse organs/tissues (e.g., the heart, kidneys, and other potential targets for radiogenic DCS) should have contributed to various aortic changes after total body irradiation (TBI). Local irradiation is, however, impractical for highly fractionated exposures and is not feasible for chronic exposures, therefore we used TBI in all irradiation regimens. Fractionated partial exposure of the mouse heart is not unfeasible reportedly, albeit at the expense of repetitive X-ray exposures posed by fluoroscopy-guided positioning [24].

### 4.3. Lesional Changes in the Aorta

We previously reported that mice defective in apolipoprotein E-deficient (ApoE^−/−^) (when fed a high-fat diet or aged), but not B6J mice (young, aged, or at 6 mos ASI), develop atherosclerotic plaques in the aorta that are positive with markers for neutral lipids (Oil Red O) and macrophages (CD68 and F4/80) and show its typical morphological features [8]. Likewise, no aortae were positive with Oil Red O in B6J mice at 12 mos ASI (representative images shown in Figure S9A), indicating that irradiation does not lead to the development of aortic atherosclerosis in B6J mice.

During the immunofluorescence and histochemical analysis of the descending thoracic aorta, we observed venous thrombus (pathological clot)-like structures in two mice at 12 mos ASI (n.b., no mice developed such structures in any of the previously reported 20 groups of B6J or ApoE^−/−^ mice [7,8]). One mouse that received 5 Gy of X-rays in 25 Frs had a structure with *x* (horizontal, major axis) = ~100 µm, *y* (horizontal, minor axis) = ~50 µm, *z* (vertical, craniocaudal axis) = ~70 µm (representative images shown in Figure S9B(a,b)). The other mouse that received chronic γ-rays had two structures in a single vessel (probably vein): one with *x* ≤ 76 µm, *y* ≤ 44 µm, *z* ≥ 60 µm, the other with *x* ≤ 36 µm, *y* ≤ 16 µm, *z* ≥ 20 µm. We are not sure whether we could observe these structures just by chance, and whether these structures are venous thrombi (e.g., positive with CD42b for platelet thrombi or phosphotungstic acid-hematoxylin for fibrin thrombi). Nonetheless, we do not rule out the possibility that irradiation promotes thrombosis, considering the radioprotective role of the platelet glycoprotein Ibα (thrombosis regulatory factor) in a mouse model on a B6J background [25].

### 4.4. Aortic Changes with Age in Non-Irradiated or Sham-Irradiated Mice

As reported previously [7,8] and here, our study included 2 non-irradiated groups (aged 8 and 104 wks) and 11 sham-irradiated groups (aged 13, 21, 34, and 60 wks) of B6J mice, each group being made up of 8–11 mice. Datasets for various endpoints were fitted to three models (linear, exponential, and logarithmic) as a function of age. Several endpoints were correlated well with age at multiple *R*-squared (*R*^2^) > 0.4, e.g., the number of crests/field (Figure S10A), IMT (Figure S10B), aortic VSMCs with subcellular fragments (Figure S10C), and intensity of aniline blue stain per unit aortic wall area (Figure S10D), suggesting that these endpoints with such age-dependent changes may have potential to serve as markers for age.

## 5. Conclusions

Our present findings suggest that irradiation of B6J mice produces vascular damage and dysfunction, inflammation, and fibrosis in the aorta, and these observed changes have all been implicated in the early stages of atherosclerosis. The integrative approaches for a series of aortic endpoints suggest that dose protraction changes aortic damage, albeit in a fashion that depends on post-irradiation time and is not a simple function of dose rate. Mechanisms behind these phenomena (e.g., cumulativeness, additivity, and synergism of damage, accelerated cellular senescence, and role of adaptive response [5]) remain unclear at this stage. Identification of such mechanistic underpinnings necessitates more investigations, which will be indispensable for considering implications for radiation oncology, radiology, and radiation protection, and for developing adverse outcome pathways and biology-based dose-response models for DCS [26,27,28,29,30,31]. With the endpoints determined in our studies to evaluate prelesional aortic changes, further studies are ongoing to examine dose protraction effects in the carotid artery as the other large artery implicated in DCS pathogenesis following radiation exposure [1,2,3].

## Data Availability

The data presented in the current study are available from the first and corresponding author (N.H.) upon reasonable request.

## Supplementary Materials

The following supporting information can be downloaded at: https://www.mdpi.com/article/10.3390/cancers14143319/s1, Footnotes to Figure 1, Figure 3, and Figure 4, Figure S1. Experimental timelines, Figure S2. Temporal changes in survival of irradiated or sham-irradiated B6J mice, Figure S3. Temporal changes in body weight of irradiated or sham-irradiated B6J mice, Figure S4. Changes in body weight, heart weight, and kidney weight at sampling, Figure S5. Echocardiographic indices for left ventricular function, Figure S6. Morphological changes in the aortic endothelium, Figure S7. Nuclear changes in the aorta, Figure S8. Fibrotic changes in the aorta, Figure S9. Lesional changes in the aorta, Figure S10. Aortic changes with age in non-irradiated or sham-irradiated mice, Table S1. List of 27 endpoints changed in irradiated mice at least in one irradiation regimen (vs sham-irradiated mice) at 12 mos ASI, Table S2. Comparison of multiple endpoints in relation to aortic changes by scoring of categorized levels of statistical differences in six pairs among four irradiation regimens, Table S3. Comparison of radiation effects in the aorta at 6 mos ASI, averaged over 15 endpoints among four irradiation regimens, Table S4. Comparison of radiation effects in the aorta at 12 mos ASI, averaged over 13 endpoints among four irradiation regimens, Table S5. Comparison of radiation effects in the aorta at 6 and 12 mos ASI, averaged over 28 endpoints among four irradiation regimens, Table S6. Comparison of all endpoints in relation to aortic changes by scoring of categorized levels of statistical differences in six pairs among four irradiation regimens, Table S7. Comparison of radiation effects in the aorta at 6 mos ASI, averaged over 20 endpoints among four irradiation regimens, Table S8. Comparison of radiation effects in the aorta at 12 mos ASI, averaged over 20 endpoints among four irradiation regimens, Table S9. Comparison of radiation effects in the aorta at 6 and 12 mos ASI, averaged over 40 endpoints among four irradiation regimens.
