# Supplementary material for: Temporal Changes in Sparing and Enhancing Dose Protraction Effects of Ionizing Irradiation for Aortic Damage in Wild-Type Mice
Source: Cancers (Basel). 2022 Jul 7;14(14):3319. doi: 10.3390/cancers14143319 (PMC9321929; doi:10.3390/cancers14143319)
Supplement: Supplementary file 1 [file cancers-14-03319-s001.zip › cancers-1751881-supplementary.pdf]

cancers

MDPI

Supplementary Materials

Temporal Changes in Sparing and Enhancing Dose Protraction Effects of Ionizing Irradiation for Aortic Damage in Wild-Type Mice

Nobuyuki Hamada, Ki-ichiro Kawano, Takaharu Nomura, Kyoji Furukawa, Farina Mohamad Yusoff, Tatsuya Maruhashi, Makoto Maeda, Ayumu Nakashima and Yukihito Higashi

Footnotes to Figures 1, 3 and 4

A footnote to Figure 1. **At 6 months after starting irradiation**, among four sham-irradiated groups, there was no heterogeneity for one endpoint (crests, $p = 0.8$, the ANOVA F-test), though such differences were untestable for the other two endpoints. Among four irradiation regimens, one endpoint (crests) was lower in three regimens (except for chronic γ-rays), and two endpoints (detachment, large detachment) were higher in two regimens (acute X-rays, X-rays in 25 fractions), in irradiated groups than in sham-irradiated groups. Among four irradiation regimens, there was heterogeneity in the degree of a difference between irradiated and sham-irradiated groups for two endpoints ($p = 1 \times 10^{-7}$ for crests, $p = 0.046$ for detachment, chi-square test), but not for one endpoint (large detachment). Among the 6 pairs of four irradiation regimens, there was an inter-regimen difference in three pairs for one endpoint (detachment) and in two pairs for two endpoints (crests, large detachment). **At 12 months after starting irradiation**, among four sham-irradiated groups, there was no difference for all three endpoints ($0.4 < p \leq 1$). Among four irradiation regimens, one endpoint (crests) was lower in two regimens (acute X-rays, X-rays in 25 fractions), in irradiated groups than in sham-irradiated groups. Among four irradiation regimens, there was heterogeneity in the degree of a difference between irradiated and sham-irradiated groups for one endpoint ($p = 0.002$ for crests, chi-square test), but not for the other two endpoints. Among the 6 pairs of four irradiation regimens, there was an inter-regimen difference in two pairs for one endpoint (crests). **For comparison at 6 and 12 months after starting irradiation**, one endpoint (crests) was higher in one regimen (X-rays in 25 fractions), and two endpoints (detachment, large detachment) were lower in two regimens (acute X-rays, X-rays in 25 fractions), at 12 months than at 6 months after starting irradiation.

A footnote to Figure 3. **At 6 months after starting irradiation**, among four irradiation regimens, four endpoints (TNF-α, CD68, CD3, and IMT) were higher in all four regimens, three endpoints (CD31 negativity, DAPI negativity, and TGF-β1) were higher in three regimens (except for chronic γ-rays), two endpoints (eNOS and VE-cadherin) were lower in three regimens (except for chronic γ-rays), and one endpoint (F4/80) was higher in three regimens (except for X-rays in 100 fractions), in irradiated groups than in sham-irradiated groups (Welch's *t*-test or Wald test). Among the 6 pairs of four irradiation regimens, there was an inter-regimen difference in four pairs for four endpoints (CD31 negativity, DAPI negativity, TNF-α, and TGF-β1), three pairs for one endpoint (VE-cadherin), and one pair for three endpoints (eNOS, CD68, and F4/80) (chi-square test). **At 12 months after starting irradiation**, among four sham-irradiated groups, there was no heterogeneity for all ten endpoints ($p > 0.11$, the ANOVA F-test). Among four irradiation regimens, three endpoints (CD31 negativity, DAPI negativity, and TNF-α) were higher in all four regimens, two endpoints (eNOS and VE-cadherin) were lower in three regimens (except for chronic γ-rays), one endpoint (TGF-β1) was higher in three regimens (except for chronic γ-rays), two endpoints (CD68 and IMT) were higher in two regimens (acute X-rays, X-rays in 25 fractions), one endpoint (CD3) was higher in two regimens (X-rays in 25 fractions, X-rays in 100 fractions), and one endpoint (F4/80) was higher in one regimen (X-rays in 25 fractions), in irradiated groups than in sham-irradiated groups (Welch's *t*-test or Wald test). Among four irradiation regimens, there was heterogeneity in the degree of a difference between irradiated and sham-irradiated groups for nine (all but TNF-α) endpoints ($p < 0.03$, chi-square test). Among the 6 pairs of four irradiation regimens, there was an inter-regimen difference in four pairs for three endpoints

(CD68, TGF-β1, and IMT), three pairs for two endpoints (eNOS and CD3), two pairs for one endpoint (VE-cadherin), and one pair for two endpoints (CD31 negativity and DAPI negativity) (chi-square test). **For comparison at 6 and 12 months after starting irradiation**, there was a difference in three regimens for three endpoints (eNOS, F4/80, and CD3), in two regimens for five endpoints (CD31 negativity, DAPI negativity, VE-cadherin, TNF-α, and TGF-β1), and in one regimen for two endpoints (CD68 and IMT) at 6 and 12 months after starting irradiation ($1 \times 10^{-6} < p < 0.1$, chi-square test).

A footnote to Figure 4B,C. **At 6 months after starting irradiation**, among four sham-irradiated groups, there was heterogeneity for two endpoints ($p < 0.02$, the ANOVA F-test). Among four irradiation regimens, one endpoint (stained intensity) was higher in all four regimens, and one endpoint (IMT) was higher in three regimens (except for chronic γ-rays), in irradiated groups than in sham-irradiated groups (Welch's *t*-test). Among four irradiation regimens, there was heterogeneity in the degree of a difference between irradiated and sham-irradiated groups for two endpoints ($p < 0.01$, chi-square test). Among the 6 pairs of four irradiation regimens, there was an inter-regimen difference in four pairs for one endpoint (stained intensity) and three pairs for one endpoint (IMT) (chi-square test). **At 12 months after starting irradiation**, among four sham-irradiated groups, there was no heterogeneity for two endpoints ($p > 0.8$, the ANOVA F-test). Among four irradiation regimens, one endpoint (stained intensity) was higher in three regimens (except for X-rays in 100 fractions), and one endpoint (IMT) was higher in one regimen (X-rays in 25 fractions), in irradiated groups than in sham-irradiated groups (Welch's *t*-test). Among four irradiation regimens, there was heterogeneity in the degree of a difference between irradiated and sham-irradiated groups for two endpoints ($p < 0.06$, chi-square test). Among the 6 pairs of four irradiation regimens, there was an inter-regimen difference in one pair for two endpoints (chi-square test). **For comparison at 6 and 12 months after starting irradiation**, there was a difference in two regimens for one endpoint (stained intensity) and in three regimens for one endpoint (IMT) at 6 and 12 months after starting irradiation ($1 \times 10^{-6} < p < 0.1$, chi-square test).

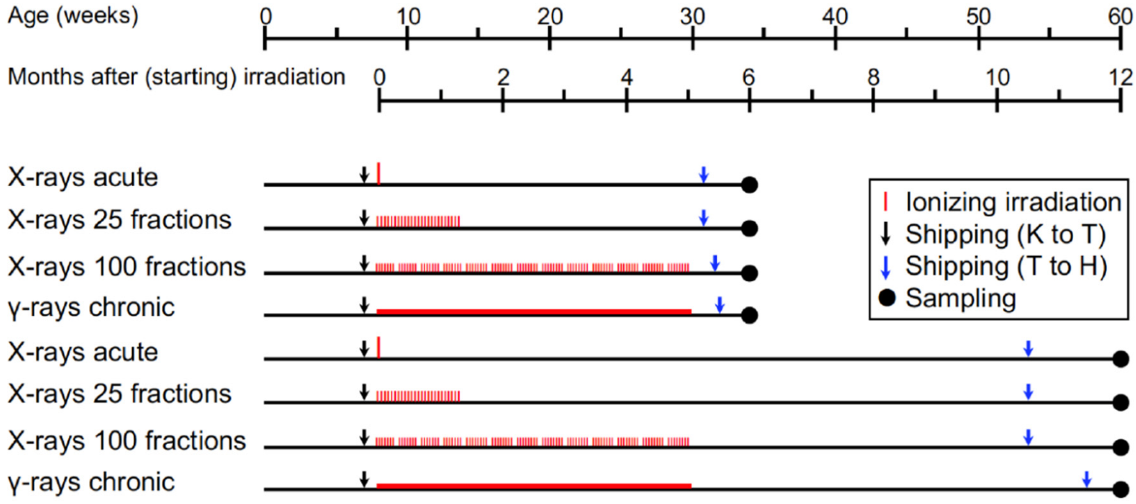

Figure S1. Experimental timelines. This study is composed of four irradiation regimens and 16 groups of wild-type C57BL6/J (B6J) male mice. For the first (upper) 8 groups that underwent sampling at 6 months after starting irradiation, the 2021 Cancers paper [1] has given details and reported the results, from which we took the data on the aorta to Figure 1A–C, Figure 3A–J, Figure 4B,C, Figures S6B, S7C(a,b), and S8A–C for comparison with the data at 12 months after starting irradiation. For the last (lower) 8 groups that underwent sampling at 12 months after starting irradiation, Section 2.1 of the main text gives details where we report the new results. K to T, Kanagawa to Tokyo (by car). T to H, Tokyo to Hiroshima (by car and air).

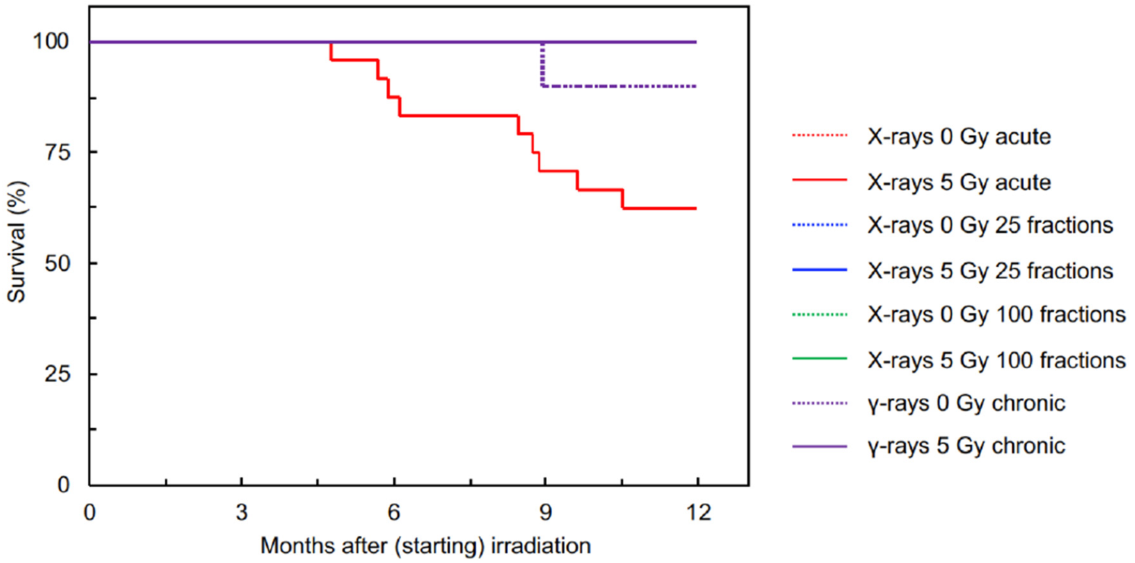

Figure S2. Temporal changes in survival of irradiated or sham-irradiated B6J mice. Mice (10 mice/group, except for 24 mice in the "X-rays 5 Gy acute" group) were irradiated or sham-irradiated at the age of 8 weeks, and survival was measured. There was no difference in survival among four sham-irradiated groups ($p > 0.32$). During the observation period, 9 out of 24 mice in the "X-rays 5 Gy acute" group and 1 out of 10 mice in the "γ-rays 0 Gy chronic" group died. There was a difference between irradiated and sham-irradiated groups in one regimen ($p = 0.033$ for acute X-rays), but not in the other three regimens ($p > 0.32$), by the log-rank test for the null hypothesis that Kaplan–Meier curves are the same.

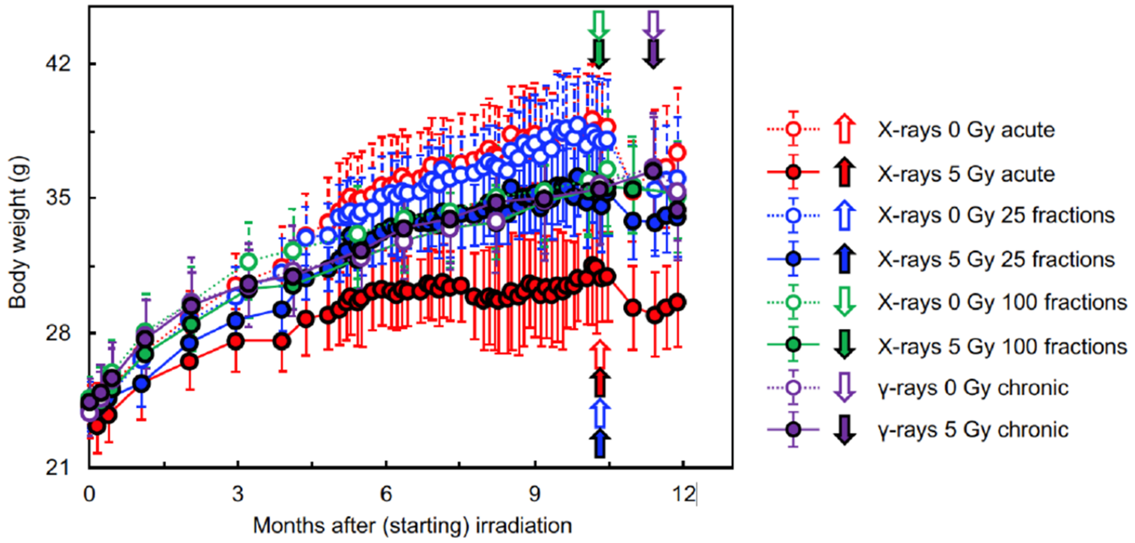

Figure S3. Temporal changes in body weight of irradiated or sham-irradiated B6J mice. Mice (10 mice/group, except for 24 mice in the "X-rays 5 Gy acute" group) were irradiated or sham-irradiated at the age of 8 weeks, and body weight was measured periodically. There was no difference in body weight among 8 groups at age 8 weeks (i.e., on day 0 when irradiation started) ($p = 0.64$, the ANOVA F-test). Except for the "X-rays 5 Gy 100 fractions" group ($p = 0.85$), shipment from Tokyo to Hiroshima (the timing indicated by arrows) led to a decrease in body weight, marginally in the "X-rays 0 Gy 100 fractions" group ($p = 0.063$) and significantly in other 6 groups ($5 \times 10^{-7} < p < 6 \times 10^{-4}$), by the one sample t-test. For the purpose of statistical analysis, the entire observation period (i.e., 0–12 months after starting irradiation) was divided into four quarters: Quarter 1 (0–3 months, 0–92), quarter 2 (3–6 months, 93–183 days), quarter 3 (6–9 months, 184–274 days), and quarter 4 (9–12 months, 275–365 days). For each quarter, a difference between irradiated and sham-irradiated groups was significant in Quarters 1–4 for the "acute X-rays" regimen ($1 \times 10^{-16} < p < 0.015$) and in Quarters 2–4 for the "X-rays in 25 fractions" regimen ($0.0016 < p < 0.026$), but not in any quarter for other two regimens ($p > 0.11$), by the t-test. Such a difference among four quarters was significant for the "acute X-rays" regimen ($p = 5 \times 10^{-26}$) and the "X-rays in 25 fractions"

regimen ($p = 3 \times 10^{-4}$), but not for the other two regimens ($p > 0.26$), by the F-test. Symbols are as indicated on the right. For comparison with the data at 6 months after starting irradiation, see the 2021 Cancers paper [1].

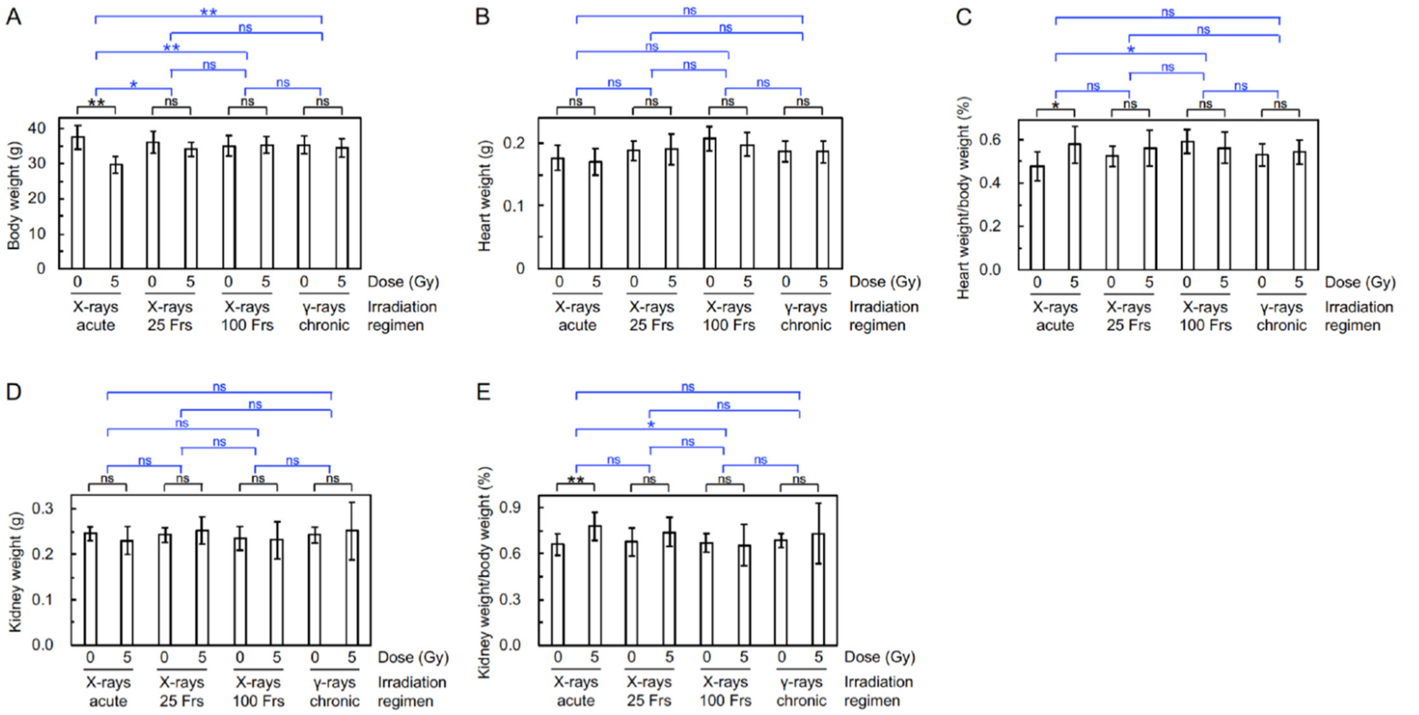

Figure S4. Changes in body weight, heart weight, and kidney weight at sampling. Mice hearts and kidneys were weighed at sampling at 12 months after starting irradiation (9–10 mice/group analyzed except for 15 mice in the "X-rays 5 Gy acute" group). (**A**) Body weight. (**B**) Heart weight. (**C**) Heart weight/body weight. (**D**) Kidney weight. (**E**) Kidney weight/body weight. Among four irradiation regimens, one endpoint (body weight) was lower, and two endpoints (heart weight/body weight, kidney weight/body weight) were higher in one regimen (acute X-rays), in irradiated groups than in sham-irradiated groups, by Welch's *t*-test. Among four irradiation regimens, there was heterogeneity in the degree of a difference between irradiated and sham-irradiated groups for two endpoints ($p = 2 \times 10^{-6}$ for body weight, and $p = 0.014$ for heart weight/body weight), but not for the other three endpoints ($p > 0.12$), by the chi-square test. Among the 6 pairs of four irradiation regimens, there was an inter-regimen difference in three pairs for one endpoint (body weight) and in one pair for two endpoints (heart weight/body weight, kidney weight/body weight). Frs, fractions. **, $p < 0.001$. *, $0.001 \leq p < 0.05$. #, $0.05 \leq p < 0.1$ (marginally significant). ns, $p \geq 0.1$ (nonsignificant). For comparison with the data at 6 months after starting irradiation, see the 2021 Cancers paper [1].

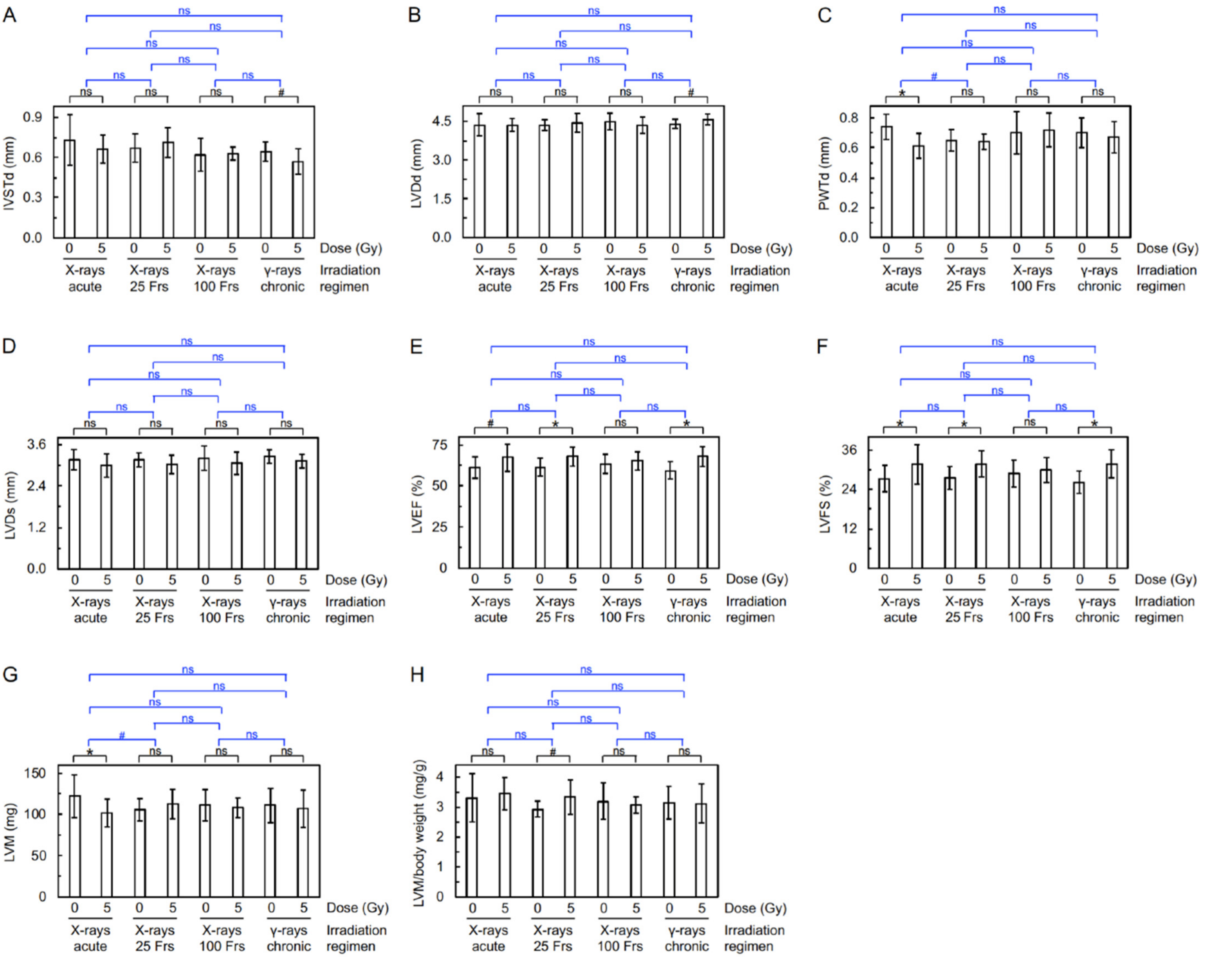

Figure S5. Echocardiographic indices for left ventricular function. Mice were subjected to M-mode echocardiography just before sampling at 12 months after starting irradiation (9–10 mice/group analyzed except for 15 mice in the "X-rays 5 Gy acute" group). (**A**) Interventricular septal thickness at end diastole (IVSTd). (**B**) Left ventricular dimension at end diastole (LVDd). (**C**) Left ventricular posterior wall thickness at end diastole (PWTd). (**D**) Left ventricular dimension at end systole (LVDs). (**E**) Left ventricular ejection fraction (LVEF). (**F**) Left ventricular fractional shortening (LVFS). (**G**) Left ventricular mass (LVM). (**H**) LVM/body weight. Among four irradiation regimens, two endpoints (LVEF, LVFS) were higher in three regimens (acute X-rays, X-rays in 25 fractions, chronic γ-rays), two endpoints (PWTd, LVM) were lower in one regimen (acute X-rays), one endpoint (LVM/body weight) was higher in one regimen (X-rays in 25 fractions), one endpoint (IVSTd) was lower and one endpoint (LVDd) was higher in one regimen (chronic γ-rays), in irradiated groups than in sham-irradiated groups, with no difference for any of eight endpoints in one regimen (X-rays in 100 fractions), by Welch's *t*-test. Among four irradiation regimens, there was heterogeneity in the degree of a difference between irradiated and sham-irradiated groups for one endpoint ($p = 0.063$ for PWTd), but not for the other seven endpoints ($p > 0.11$), by the chi-square test. Among the 6 pairs of four irradiation regimens, there was an inter-regimen difference in one pair for two endpoints (PWTd, LVM). Frs, fractions. **, $p < 0.001$. *, $0.001 \le p < 0.05$. #, $0.05 \le p < 0.1$ (marginally significant). ns, $p \ge 0.1$ (nonsignificant). For comparison with the data at 6 months after starting irradiation, see the 2021 Cancers paper [1].

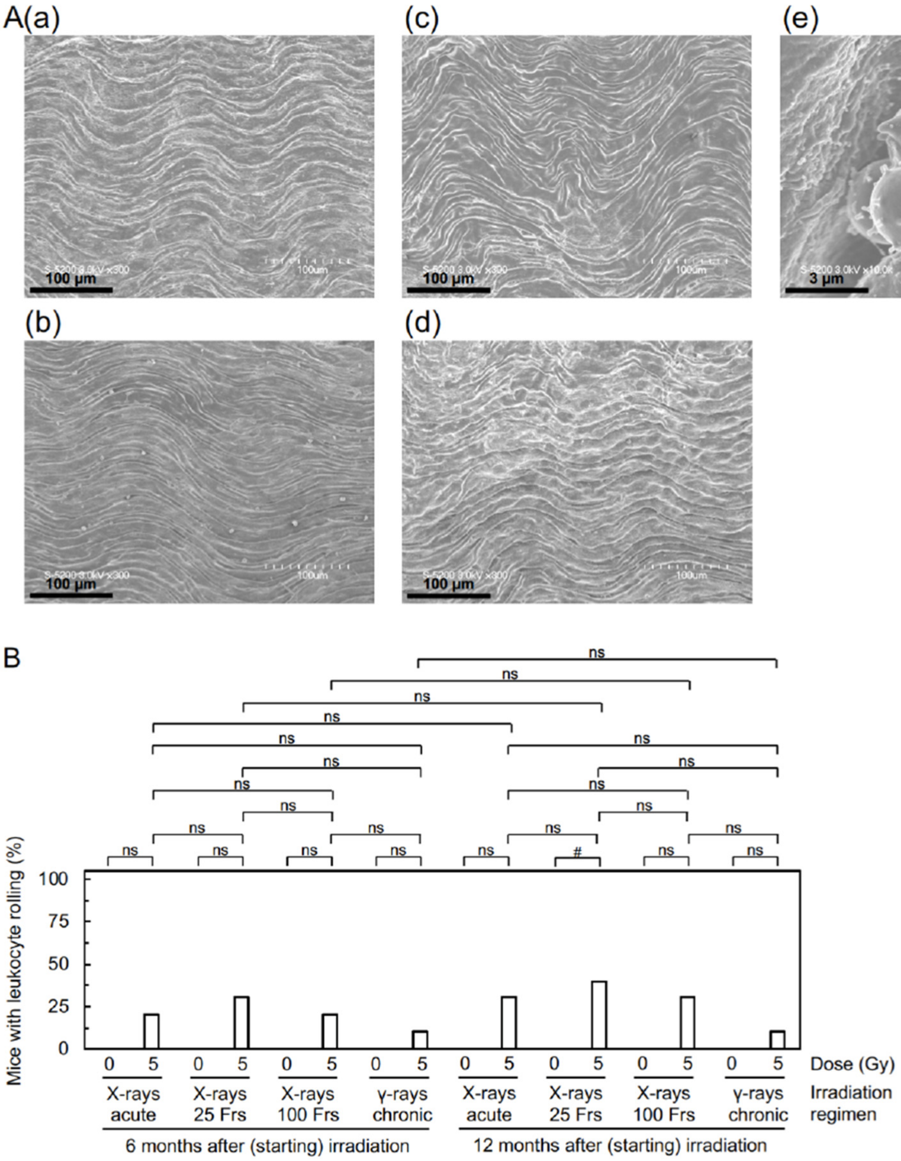

Figure S6. Morphological changes in the aortic endothelium. (**A**) Representative FE-SEM images of (**a**) normal endothelium, (**b**) flattened endothelium, (**c**) deranged endothelium, (**d**) cobblestone (or snap pea)-shaped endothelium, and (**e**) endothelium with rolling leukocytes. (**a**) X-rays 0 Gy acute, (**b**) X-rays 5 Gy acute, and (**c**–**e**) X-rays 5 Gy 25 fractions, all at 12 months after starting irradiation. Scale bars are as indicated. For representative images of detachment and large detachment, see the 2020 and 2021 Cancers papers [1,2]. (**B**) Quantitative analysis for percentage of mice with leukocyte rolling (8–10 mice/group analyzed, Fisher's exact test). There was no inter-group difference in any comparisons, except for a marginal difference ($p = 0.087$) in one regimen (X-rays in 25 fractions) at 12 months after starting irradiation. Frs, fractions. #, $0.05 \leq p < 0.1$ (marginally significant). ns, $p \geq 0.1$ (nonsignificant). The data in Figure S6B for 8 groups at 6 months after starting irradiation were taken from the 2021 Cancers paper [1].

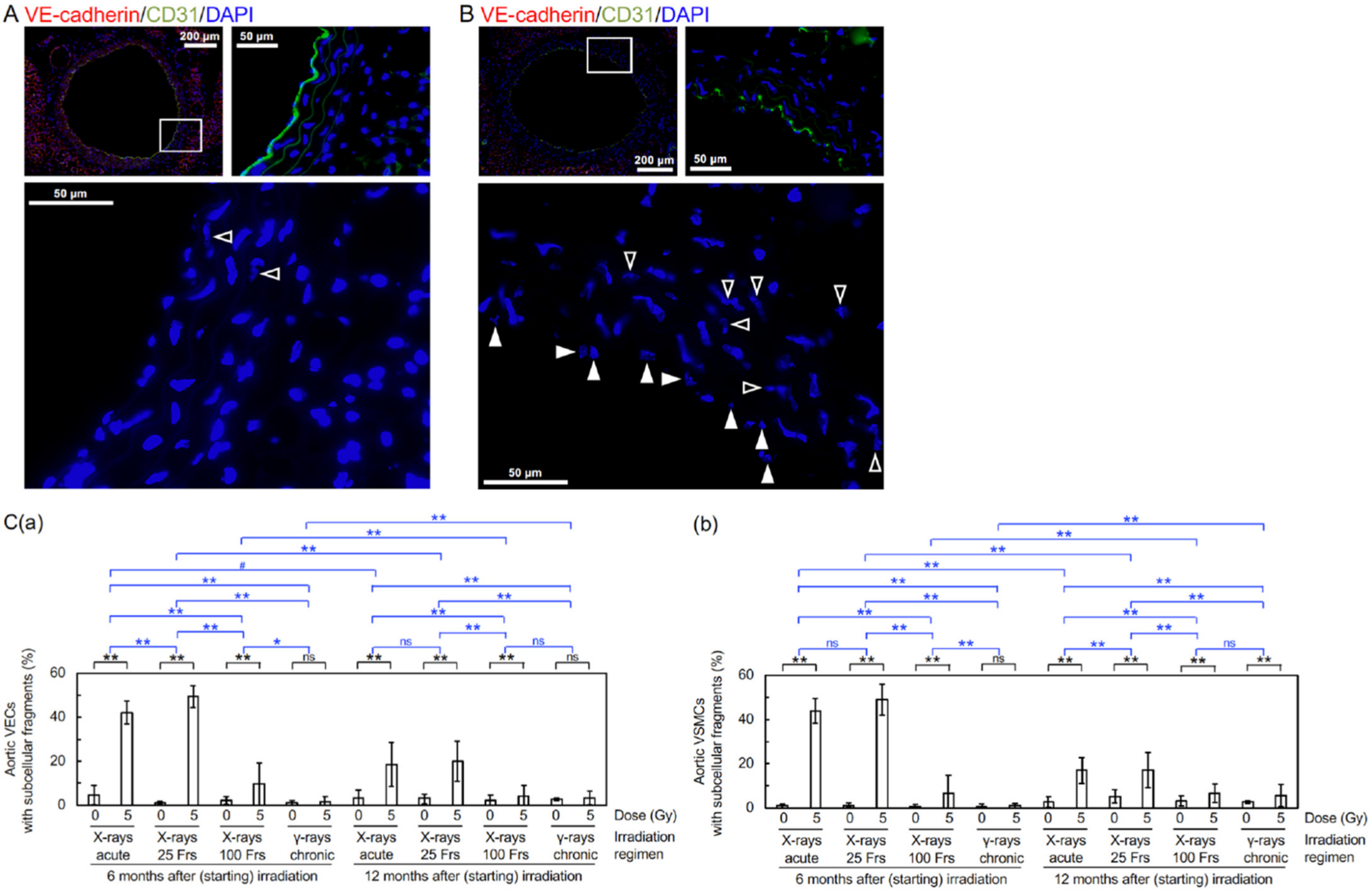

Figure S7. Nuclear changes in the aorta. (**A**,**B**). Representative immunofluorescence images for sham (0 Gy)-irradiated (**A**) or 5 Gy-irradiated (**B**) cells with subcellular fragments in the aorta. Boxed areas in the upper left panels (tiled images) are shown at higher magnification in the upper right panels. The upper left panel in Figure S7A is the same as the upper left panel in Figure 2B (i.e., at 12 months after starting irradiation with 0 Gy of acute X-rays): likewise, the upper right panel is the same as the upper right panel in Figure 2B, but without VE-cadherin (i.e., the image merged only for CD31 and DAPI). The upper left panel in Figure S7B is the same as the lower left panel in Figure 2B (i.e., at 12 months after starting irradiation with 5 Gy of acute X-rays): likewise, the upper right panel is the same as the lower right panel in Figure 2B, but without VE-cadherin (i.e., the image merged only for CD31 and DAPI). The upper right panels are further enlarged in the lower panels, but without CD31 (i.e., only DAPI). Closed and open arrowheads point to vascular endothelial cells (VECs) with subcellular fragments in the tunica intima and vascular smooth muscle cells (VSMCs) with subcellular fragments in the tunica media, respectively. Scale bars are as indicated. (**C**). Quantitative analysis for (**a**) aortic VECs with subcellular fragments and (**b**) aortic VSMCs with subcellular fragments (8–10 mice/group analyzed, 133–387 VECs and 407–1670 VSMCs counted/mouse). (**a**,**b**) **At 6 months after starting irradiation**, among four irradiation regimens, two endpoints were higher in three regimens (except for chronic γ-rays), in irradiated groups than in sham-irradiated groups (Wald test). Among the 6 pairs of four irradiation regimens, there was an inter-regimen difference in all six pairs for one endpoint (VECs) and five pairs for one endpoint (VSMCs) (chi-square test). **At 12 months after starting irradiation**, among four sham-irradiated groups, there was heterogeneity for two endpoints ($p < 0.01$, the ANOVA F-test). Among four irradiation regimens, one endpoint (VECs) was higher in three regimens (except for chronic γ-rays), and one endpoint (VSMCs) was higher in four regimens, in irradiated groups than in sham-irradiated groups (Wald test). Among four irradiation regimens, there was heterogeneity in the degree of a difference between irradiated and sham-irradiated groups for two endpoints ($p < 3 \times 10^{-17}$, chi-square test). Among the 6 pairs of four irradiation regimens, there was an inter-regimen difference in four pairs for one endpoint (VECs) and five pairs for one endpoint (VSMCs) (chi-square test). **For comparison at 6 and 12 months after starting irradiation**, there was a difference in four regimens for two endpoints at 6 and 12 months after starting irradiation ($7 \times 10^{-18} < p < 0.06$, chi-square test). Frs, fractions. **, $p < 0.001$. *, $0.001 \leq p < 0.05$. #, $0.05 \leq p < 0.1$ (marginally significant). ns, $p \geq 0.1$ (nonsignificant). The data in Figure S7C(a,b) for 8 groups at 6 months after starting irradiation were taken from the 2021 Cancers paper [1].

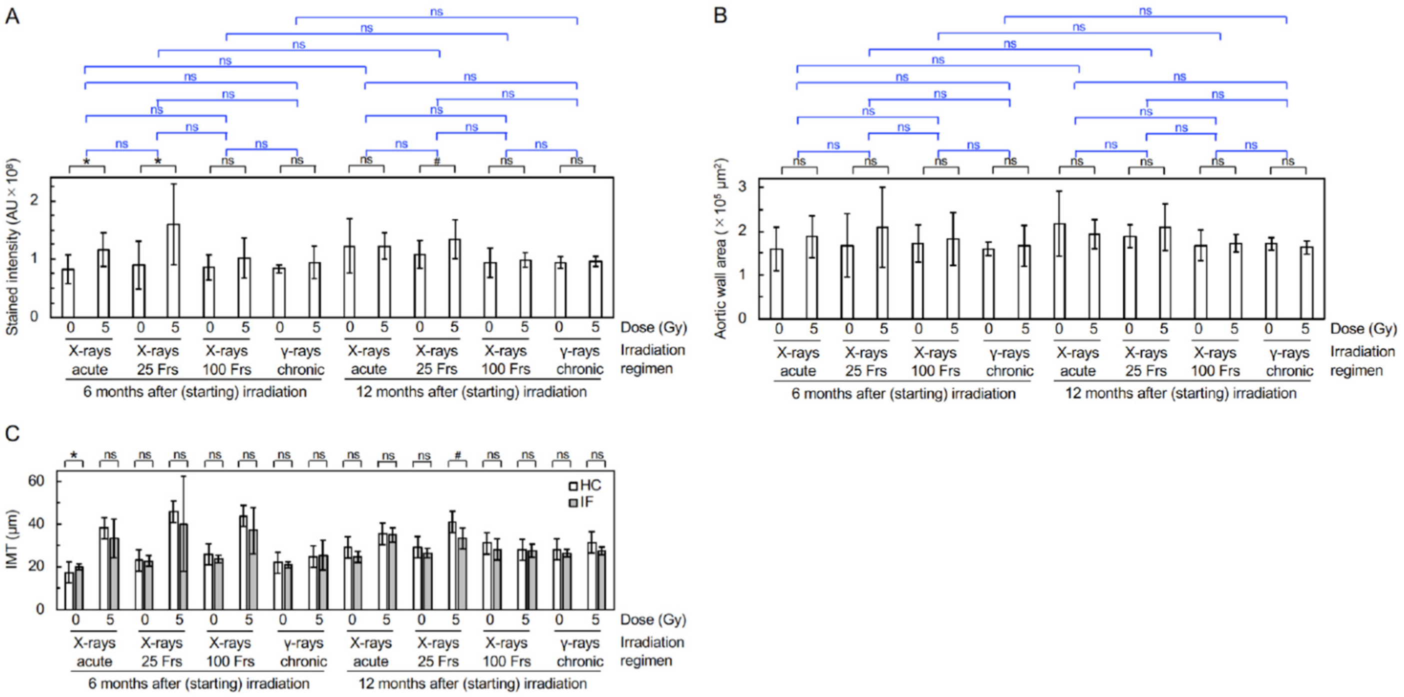

Figure S8. Fibrotic changes in the aorta. Quantitative analysis for (**A**) total intensity of aniline blue stain in the entire aortic wall, and (**B**) aortic wall area (9–10 mice/group analyzed). AU, arbitrary unit. **At 6 months after starting irradiation**, among four sham-irradiated groups, there was no heterogeneity for two endpoints ($p > 0.9$, the ANOVA F-test). Among four irradiation regimens, one endpoint (total intensity) was higher in two regimens (acute X-rays, X-rays in 25 fractions), in irradiated groups than in sham-irradiated groups (Welch's *t*-test). Among four irradiation regimens, there was no heterogeneity in the degree of a difference between irradiated and sham-irradiated groups for two endpoints ($p > 0.3$, chi-square test). There was no inter-regimen difference in any of the 6 pairs of four irradiation regimens for two endpoints (chi-square test). **At 12 months after starting irradiation**, among four sham-irradiated groups, there was marginal heterogeneity for one endpoint (aortic wall area, $p = 0.07$, the ANOVA F-test), but not for the other endpoint. Among four irradiation regimens, one endpoint (total intensity) was higher in one regimen (X-rays in 25 fractions), in irradiated groups than in sham-irradiated groups (Welch's *t*-test). Among four irradiation regimens, there was no heterogeneity in the degree of a difference between irradiated and sham-irradiated groups for two endpoints ($p > 0.3$, chi-square test). There was no inter-regimen difference in any of the 6 pairs of four irradiation regimens for two endpoints (chi-square test). **For comparison at 6 and 12 months after starting irradiation**, there was no difference in any of the four irradiation regimens for two endpoints at 6 and 12 months after starting irradiation ($0.1 < p < 0.9$, chi-square test). (C). Comparison of IMT determined from histochemistry images (open columns) or from immunofluorescence images (filled columns). Replotted from Figure 3J and Figure 4C (see its legend for details). There was no difference in IMT between any of the 16 groups except for two groups (Welch's *t*-test). Frs, fractions. *, $0.001 \leq p < 0.05$. #, $0.05 \leq p < 0.1$ (marginally significant). ns, $p \geq 0.1$ (nonsignificant). The data in Figure S8A–C for 8 groups at 6 months after starting irradiation were taken from the 2021 Cancers paper [1].

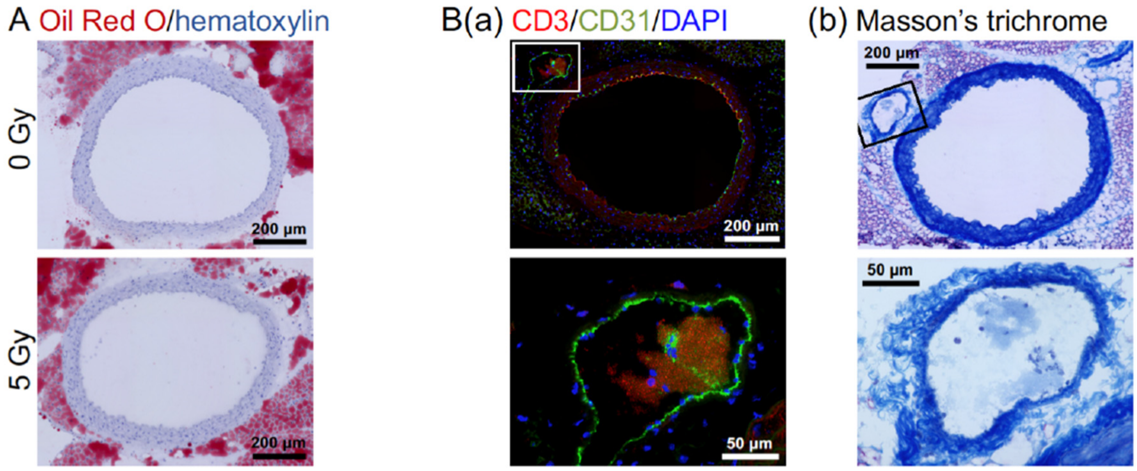

Figure S9. Lesional changes in the aorta. (**A**) Representative images for Oil Red O staining (at 12 months after starting irradiation with 0 Gy or 5 Gy of X-rays in 25 fractions). For representative images of the Oil Red O-positive aorta in mice, see Figure S10B of the 2021 Cancers paper [1]. (**B**) Representative images for a venous thrombus-like structure (at 12 months after starting irradiation with 5 Gy of X-rays in 25 fractions). (**a**) The aorta was subjected to double immunofluorescence of CD3 and CD31, with cell nuclei counterstained with DAPI. (**b**) The aorta was subjected to Masson's trichrome staining. (**a**,**b**) A boxed area in the upper panel (a tiled image) is shown at higher magnification in the lower panel. Scale bars are as indicated.

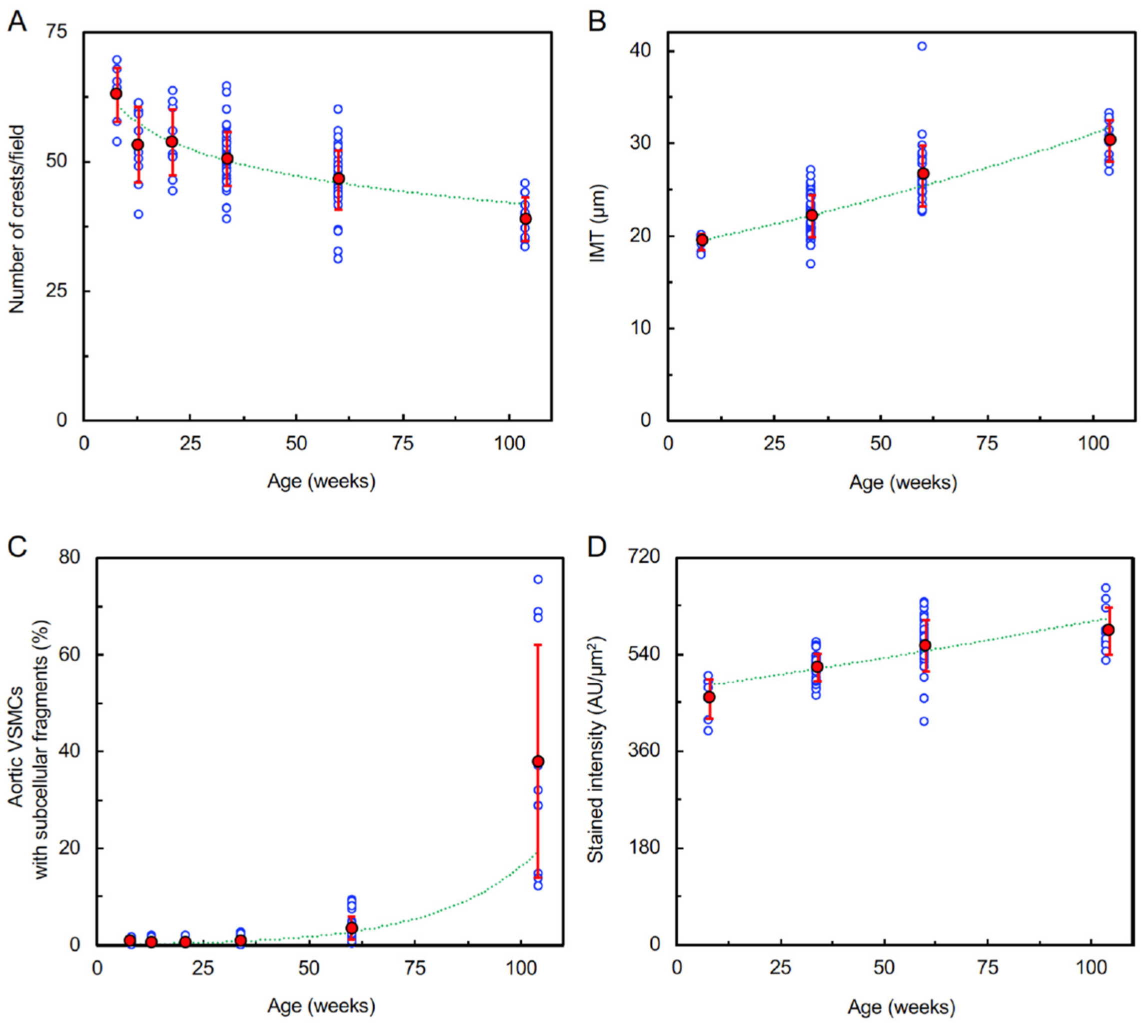

Figure S10. Aortic changes with age in non-irradiated or sham-irradiated mice. Each datapoint consists of 7–8 mice at age 8 weeks (from 1 non-irradiated group reported in [2]), 10 mice at age 13 weeks (from 1 sham-irradiated group reported in [2]), 10 mice at age 21 weeks (from 1 sham-irradiated group reported in [2]), 40–48 mice at age 34 weeks (from 4–5 sham-irradiated groups reported in [1,2]), 35–39 mice at age 60 weeks (from 4 sham-irradiated groups reported here), and 9–10 mice at age 104 weeks (from 1 non-irradiated group reported in [1]). Blue open circles, data from each mouse. Red closed circles and error bars, means and standard deviations at each age. Datasets for each endpoint were fitted to three models (linear, exponential, logarithmic), where y is the number of crests/fields and x is age in weeks. R^2, multiple R-squared. p, by the F-test. (**A**) Alterations in the number of crests/fields. All three models fitted the data well, with a logarithmic model (a green dotted line, $y = 76.4453–7.4575*1n(x)$, $R^2 = 0.4190$, $p = 5 \times 10^{-16}$) slightly better than an exponential model ($y = 58.4776*e^{-0.004030*x}$, $R^2 = 0.4045$, $p = 2 \times 10^{-15}$) or a linear model ($y = 58.1286–0.1950*x$, $R^2 = 0.3996$, $p = 3 \times 10^{-15}$). (**B**) Alterations in IMT determined from immunofluorescence images. All three models fitted the data well, with an exponential model (a green dotted line, $y = 18.7691*e^{-0.005051*x}$, $R^2 = 0.5805$, $p = 3 \times 10^{-20}$) slightly better than a linear model ($y = 18.1980+0.1256*x$, $R^2 = 0.5666$, $p = 2 \times 10^{-19}$) or a logarithmic model ($y = 6.5061+4.7525*1n(x)$, $R^2 = 0.4852$, $p = 9 \times 10^{-16}$). (**C**) Alterations in aortic VSMCs with subcellular fragments. All three models fitted the data well, with an exponential model (a green dotted line, $y = 0.09715*e^{-0.05269*x}$, $R^2 = 0.5674$, $p = 2 \times 10^{-16}$) better than a linear model ($y = –9.5064+0.3289*x$, $R^2 = 0.4499$, $p = 5 \times 10^{-17}$) or a logarithmic model ($y = –27.3137+8.9290*1n(x)$, $R^2 = 0.2237$, $p = 5 \times 10^{-8}$). (**D**) Alterations in intensity of aniline blue stain per unit aortic wall area. All three models fitted the data well, with a logarithmic model (a green dotted line, $y = 338.3272+52.5070*1n(x)$, $R^2 = 0.4195$, $p = 8 \times 10^{-13}$) slightly better than a linear model ($y = 472.1662+1.2740*x$, $R^2 = 0.3836$, $p = 1 \times 10^{-11}$) or an exponential model ($y = 473.4033*e^{0.002389*x}$, $R^2 = 0.3727$, $p = 3 \times 10^{-11}$).

Supplementary Tables

Table S1. List of 27 endpoints changed in irradiated mice at least in one irradiation regimen (vs sham-irradiated mice) at 12 months after starting irradiation.

20 endpoints increased	
	Heart weight/body weight, kidney weight/body weight, LVDd, LVEF, LVFS, LVM/body weight, mice with leukocyte rolling, CD31 negativity, DAPI negativity, TNF-α, CD68, F4/80, CD3, TGF-β1, IMT-IF, aortic VECs with subcellular fragments, aortic VSMCs with subcellular fragments, stained intensity per unit aortic wall area, total stained intensity in the entire aortic wall area, IMT-HC
7 endpoints decreased	
	Body weight, IVSTd, PWTd, LVM, the number of crests/fields, eNOS, VE-cadherin

IMT, intima-media thickness. IMT-IF, IMT determined from immunofluorescence images. IMT-HC, IMT determined from histochemistry images. For details of statistical comparisons in each endpoint, see the respective figure and its legend (Figure 1, Figure 3, Figure 4, and Figures S4–S8). For endpoints changed at 6 months after starting irradiation, see Table S1 in the 2021 Cancers paper [1].

Table S2. Comparison of multiple endpoints in relation to aortic changes by scoring of categorized levels of statistical differences in six pairs among four irradiation regimens.

Months after starting irradiation	Endpoint	Scores for differences among irradiation regimens[a]					
		X-rays 25 Frs vs acute X-rays	Acute X-rays vs X-rays 100 Frs	Acute X-rays vs chronic γ-rays	X-rays 25 Frs vs X-rays 100 Frs	X-rays 25 Frs vs chronic γ-rays	X-rays 100 Frs vs chronic γ-rays
6[b]	The number of crests/fields	0.0	0.0	0.0	1.5	1.5	0.0
	Mice with detachment	0.0	1.0	1.0	1.0	1.5	0.0
	Mice with large detachment	0.0	0.0	0.0	1.5	1.5	0.0
	CD31 negativity	0.0	0.0	1.5	1.0	1.5	1.5
	DAPI negativity	0.0	0.0	1.5	1.0	1.5	1.5
	eNOS	0.0	0.0	1.0	0.0	0.0	0.0
	VE-cadherin	0.0	0.0	1.5	0.0	1.5	1.5
	TNF-α	0.0	1.5	1.5	1.0	1.5	0.0
	CD68	0.0	0.0	0.0	0.0	1.0	0.0
	F4/80	0.0	0.0	0.0	1.0	0.0	0.0
	TGF-β1	0.5	0.0	1.0	0.0	1.5	1.0
	Aortic VECs with subcellular fragments	1.5	1.5	1.5	1.5	1.5	1.0
	Aortic VSMCs with subcellular fragments	0.0	1.5	1.5	1.5	1.5	1.5

	Stained intensity per unit aortic wall area	1.5	0.0	1.0	1.5	1.5	0.0
	IMT-HC	0.0	0.0	1.0	0.0	1.0	0.5
12[c]	The number of crests/fields	0.0	0.0	1.5	0.0	1.0	0.0
	CD31 negativity	0.0	0.0	0.0	1.0	0.0	0.0
	DAPI negativity	0.0	0.0	0.0	1.0	0.0	0.0
	eNOS	0.0	0.0	1.0	0.0	1.0	0.5
	VE-cadherin	0.0	0.0	0.0	1.5	1.5	0.0
	CD68	0.0	1.0	1.0	1.0	1.0	0.0
	CD3	1.0	0.0	0.0	1.0	1.5	0.0
	TGF-β1	0.0	1.0	1.5	0.0	1.5	1.0
	IMT-IF	0.0	1.5	1.5	1.0	1.0	0.0
	Aortic VECs with subcellular fragments	0.0	1.5	1.5	1.5	1.5	0.0
	Aortic VSMCs with subcellular fragments	1.5	1.5	1.5	1.5	1.5	0.0
	Stained intensity per unit aortic wall area	0.0	1.0	0.0	0.0	0.0	0.0
	IMT-HC	0.0	0.0	0.0	0.5	0.0	0.0
6	Mean score[d]	0.2	0.4	0.9	0.8	1.2	0.6
	Difference judged from each comparison[e]	X-rays 25 Frs	Acute X-rays	Acute X-rays	X-rays 25 Frs	X-rays 25 Frs	X-rays 100 Frs
		> acute X-rays	> X-rays 100 Frs	>> chronic γ-rays	>> X-rays 100 Frs	>> chronic γ-rays	>> chronic γ-rays
	Overall difference judged from all comparisons[f]	X-rays 25 Frs > acute X-rays > X-rays 100 Frs >> chronic γ-rays					
12	Mean score[g]	0.2	0.6	0.7	0.8	0.9	0.1
	Difference judged from each comparison[e]	X-rays 25 Frs	Acute X-rays	Acute X-rays	X-rays 25 Frs	X-rays 25 Frs	X-rays 100 Frs
		≥ acute X-rays	>> X-rays 100 Frs	>> chronic γ-rays	>> X-rays 100 Frs	>> chronic γ-rays	≥ chronic γ-rays
	Overall difference judged from all comparisons[f]	X-rays 25 Frs ≥ acute X-rays >> X-rays 100 Frs ≥ chronic γ-rays					
6 and 12	Mean score[h]	0.2	0.5	0.8	0.8	1.1	0.4
	Difference judged from each comparison[e]	X-rays 25 Frs	Acute X-rays	Acute X-rays	X-rays 25 Frs	X-rays 25 Frs	X-rays 100 Frs

	> acute X-rays	> X-rays 100 Frs	>> chronic γ-rays	>> X-rays 100 Frs	>> chronic γ-rays	> chronic γ-rays
Overall difference judged from all comparisons[f]	X-rays 25 Frs > acute X-rays > X-rays 100 Frs > chronic γ-rays					

Fr, fractions. IMT, intima-media thickness. IMT-IF, IMT determined from immunofluorescence images. IMT-HC, IMT determined from histochemistry images. [a] Criteria for "A vs B" comparisons: ** = 1.5 (A > B), * = 1 (A > B), # = 0.5 (A > B), ns = 0 (A = B). [b] The data for 5 out of 20 endpoints (mice with leukocyte rolling, CD3, IMT-IF, total stained intensity in the entire aortic wall area, and aortic wall area) were omitted due to the lack of significant differences in any of the 6 pairs among four irradiation regimens. [c] The data for 7 out of 20 endpoints (mice with detachment, mice with large detachment, mice with leukocyte rolling, TNF-α, F4/80, total stained intensity in the entire aortic wall area, and aortic wall area) were omitted due to the lack of significant differences in any of the 6 pairs among four irradiation regimens. [d] Mean score ranged from 0.23 (X-rays 25 Frs vs acute X-rays) to 1.23 (X-rays 25 Frs vs chronic γ-rays). [e] Criteria for mean scores: >> (mean score ≥ 0.5), > (0.2 ≤ mean score < 0.5), ≥ (0.1 ≤ mean score < 0.2). [f] Judged from differences in each of 6 comparisons shown immediately above. [g] Mean score ranged from 0.12 (X-rays 100 Frs vs chronic γ-rays) to 0.88 (X-rays 25 Frs vs chronic γ-rays). [h] Mean score ranged from 0.21 (X-rays 25 Frs vs acute X-rays) to 1.07 (X-rays 25 Frs vs chronic γ-rays).

Table S3. Comparison of radiation effects in the aorta at 6 months after starting irradiation, averaged over 15 endpoints among four irradiation regimens.

Alternative hypotheses[a]	One-sided *p* values for equal averaged effects between the given irradiation conditions A vs B[b]					
	X-rays 25 Frs vs acute X-rays	Acute X-rays vs X-rays 100 Frs	Acute X-rays vs chronic γ-rays	X-rays 25 Frs vs X-rays 100 Frs	X-rays 25 Frs vs chronic γ-rays	X-rays 100 Frs vs chronic γ-rays
|A| > |B|	3×10^{-2}	2×10^{-8}	0×10^{0}	2×10^{-10}	0×10^{0}	1×10^{-6}
|A| < |B|	8×10^{-1}	1×10^{0}	1×10^{0}	1×10^{0}	1×10^{0}	1×10^{0}
Difference in each comparison[c]	X-rays 25 Frs > acute X-rays	Acute X-rays >> X-rays 100 Frs	Acute X-rays >> chronic γ-rays	X-rays 25 Frs >> X-rays 100 Frs	X-rays 25 Frs >> chronic γ-rays	X-rays 100 Frs >> chronic γ-rays
Overall difference[d]	X-rays 25 Frs > acute X-rays >> X-rays 100 Frs >> chronic γ-rays					

Frs, fractions. [a] vs null hypothesis that the absolute values of the radiation effects between the given comparison groups are equal for all endpoints (\|A\| = \|B\|). [b] *p*-value of one-sided Kolmogorov–Smirnov goodness-of-fit test for the null hypothesis that the distribution of the *p* values from the individual tests (in Table S2) is the standard uniform, which is true with no difference in radiation effects for all endpoints between the given comparison groups. [c] Criteria for *p* values for \|A\| > \|B\|: >> ($p < 1 \times 10^{-5}$), > ($1 \times 10^{-5} \le p < 1 \times 10^{-1}$). [d] Judged from differences in each of 6 comparisons shown immediately above.

Table S4. Comparison of radiation effects in the aorta at 12 months after starting irradiation, averaged over 13 endpoints among four irradiation regimens.

Alternative hypotheses[a]	One-sided *p* values for equal averaged effects between the given irradiation conditions A vs B[b]					
	X-rays 25 Frs vs acute X-rays	Acute X-rays vs X-rays 100 Frs	Acute X-rays vs chronic γ-rays	X-rays 25 Frs vs X-rays 100 Frs	X-rays 25 Frs vs chronic γ-rays	X-rays 100 Frs vs chronic γ-rays
|A| > |B|	1×10^{-1}	3×10^{-10}	4×10^{-8}	6×10^{-12}	1×10^{-13}	2×10^{-1}
|A| < |B|	8×10^{-1}	1×10^{0}	1×10^{0}	1×10^{0}	1×10^{0}	7×10^{-1}
Difference in each comparison[c]	X-rays 25 Frs	Acute X-rays	Acute X-rays	X-rays 25 Frs	X-rays 25 Frs	X-rays 100 Frs

	≥ acute X-rays	>> X-rays 100 Frs	>> chronic γ-rays	>> X-rays 100 Frs	>> chronic γ-rays	≥ chronic γ-rays
Overall difference[d]	X-rays 25 Frs ≥ acute X-rays >> X-rays 100 Frs ≥ chronic γ-rays					

Frs, fractions. [a] vs null hypothesis that the absolute values of the radiation effects between the given comparison groups are equal for all endpoints (|A| = |B|). [b] *p*-value of one-sided Kolmogorov–Smirnov goodness-of-fit test for the null hypothesis that the distribution of the *p* values from the individual tests (in Table S2) is the standard uniform, which is true with no difference in radiation effects for all endpoints between the given comparison groups. [c] Criteria for *p* values for |A| > |B|: >> ($p < 1 \times 10^{-5}$), > ($1 \times 10^{-5} \leq p < 1 \times 10^{-1}$), ≥ ($1 \times 10^{-1} \leq p < 3 \times 10^{-1}$). [d] Judged from differences in each of the 6 comparisons shown immediately above.

Table S5. Comparison of radiation effects in the aorta at 6 and 12 months after starting irradiation, averaged over 28 endpoints among four irradiation regimens.

Alternative hypotheses[a]	One-sided *p* values for equal averaged effects between the given irradiation conditions A vs B[b]					
	X-rays 25 Frs vs acute X-rays	Acute X-rays vs X-rays 100 Frs	Acute X-rays vs chronic γ-rays	X-rays 25 Frs vs X-rays 100 Frs	X-rays 25 Frs vs chronic γ-rays	X-rays 100 Frs vs chronic γ-rays
|A| > |B|	2×10^{-2}	0×10^{0}	0×10^{0}	0×10^{0}	0×10^{0}	4×10^{-5}
|A| < |B|	7×10^{-1}	1×10^{0}	1×10^{0}	1×10^{0}	1×10^{0}	9×10^{-1}
Difference in each comparison[c]	X-rays 25 Frs > acute X-rays	Acute X-rays >> X-rays 100 Frs	Acute X-rays >> chronic γ-rays	X-rays 25 Frs >> X-rays 100 Frs	X-rays 25 Frs >> chronic γ-rays	X-rays 100 Frs > chronic γ-rays
Overall difference[d]	X-rays 25 Frs > acute X-rays >> X-rays 100 Frs > chronic γ-rays					

Frs, fractions. [a] vs null hypothesis that the absolute values of the radiation effects between the given comparison groups are equal for all endpoints (|A| = |B|). [b] *p*-value of one-sided Kolmogorov–Smirnov goodness-of-fit test for the null hypothesis that the distribution of the *p* values from the individual tests (in Table S2) is the standard uniform, which is true with no difference in radiation effects for all endpoints between the given comparison groups. [c] Criteria for *p* values for |A| > |B|: >> ($p < 1 \times 10^{-5}$), > ($1 \times 10^{-5} \leq p < 1 \times 10^{-1}$). [d] Judged from differences in each of the 6 comparisons shown immediately above.

Table S6. Comparison of all endpoints in relation to aortic changes by scoring of categorized levels of statistical differences in six pairs among four irradiation regimens.

Months after starting irradiation	Endpoint	Scores for differences among irradiation regimens[a]					
		X-rays 25 Frs vs acute X-rays	Acute X-rays vs X-rays 100 Frs	Acute X-rays vs chronic γ-rays	X-rays 25 Frs vs X-rays 100 Frs	X-rays 25 Frs vs chronic γ-rays	X-rays 100 Frs vs chronic γ-rays
6	The number of crests/fields	0.0	0.0	0.0	1.5	1.5	0.0
	Mice with detachment	0.0	1.0	1.0	1.0	1.5	0.0
	Mice with large detachment	0.0	0.0	0.0	1.5	1.5	0.0
	Mice with leukocyte rolling	0.0	0.0	0.0	0.0	0.0	0.0
	CD31 negativity	0.0	0.0	1.5	1.0	1.5	1.5
	DAPI negativity	0.0	0.0	1.5	1.0	1.5	1.5

	eNOS	0.0	0.0	1.0	0.0	0.0	0.0
	VE-cadherin	0.0	0.0	1.5	0.0	1.5	1.5
	TNF-α	0.0	1.5	1.5	1.0	1.5	0.0
	CD68	0.0	0.0	0.0	0.0	1.0	0.0
	F4/80	0.0	0.0	0.0	1.0	0.0	0.0
	CD3	0.0	0.0	0.0	0.0	0.0	0.0
	TGF-β1	0.5	0.0	1.0	0.0	1.5	1.0
	IMT-IF	0.0	0.0	0.0	0.0	0.0	0.0
	Aortic VECs with subcellular fragments	1.5	1.5	1.5	1.5	1.5	1.0
	Aortic VSMCs with subcellular fragments	0.0	1.5	1.5	1.5	1.5	1.5
	Stained intensity per unit aortic wall area	1.5	0.0	1.0	1.5	1.5	0.0
	Total stained intensity in the entire aortic wall area	0.0	0.0	0.0	0.0	0.0	0.0
	Aortic wall area	0.0	0.0	0.0	0.0	0.0	0.0
	IMT-HC	0.0	0.0	1.0	0.0	1.0	0.5
12	The number of crests/fields	0.0	0.0	1.5	0.0	1.0	0.0
	Mice with detachment	0.0	0.0	0.0	0.0	0.0	0.0
	Mice with large detachment	0.0	0.0	0.0	0.0	0.0	0.0
	Mice with leukocyte rolling	0.0	0.0	0.0	0.0	0.0	0.0
	CD31 negativity	0.0	0.0	0.0	1.0	0.0	0.0
	DAPI negativity	0.0	0.0	0.0	1.0	0.0	0.0
	eNOS	0.0	0.0	1.0	0.0	1.0	0.5
	VE-cadherin	0.0	0.0	0.0	1.5	1.5	0.0
	TNF-α	0.0	0.0	0.0	0.0	0.0	0.0
	CD68	0.0	1.0	1.0	1.0	1.0	0.0
	F4/80	0.0	0.0	0.0	0.0	0.0	0.0
	CD3	1.0	0.0	0.0	1.0	1.5	0.0
	TGF-β1	0.0	1.0	1.5	0.0	1.5	1.0

	IMT-IF	0.0	1.5	1.5	1.0	1.0	0.0
	Aortic VECs with subcellular fragments	0.0	1.5	1.5	1.5	1.5	0.0
	Aortic VSMCs with subcellular fragments	1.5	1.5	1.5	1.5	1.5	0.0
	Stained intensity per unit aortic wall area	0.0	1.0	0.0	0.0	0.0	0.0
	Total stained intensity in the entire aortic wall area	0.0	0.0	0.0	0.0	0.0	0.0
	Aortic wall area	0.0	0.0	0.0	0.0	0.0	0.0
	IMT-HC	0.0	0.0	0.0	0.5	0.0	0.0
6	Mean score[b]	0.2	0.3	0.7	0.6	0.9	0.4
	Difference judged from each comparison[c]	X-rays 25 Frs ≥ acute X-rays	Acute X-rays > X-rays 100 Frs	Acute X-rays >> chronic γ-rays	X-rays 25 Frs >> X-rays 100 Frs	X-rays 25 Frs >> chronic γ-rays	X-rays 100 Frs > chronic γ-rays
	Overall difference judged from all comparisons[d]	X-rays 25 Frs ≥ acute X-rays > X-rays 100 Frs > chronic γ-rays					
12	Mean score[e]	0.1	0.4	0.5	0.5	0.6	0.1
	Difference judged from each comparison[c]	X-rays 25 Frs ≥ acute X-rays	Acute X-rays > X-rays 100 Frs	Acute X-rays > chronic γ-rays	X-rays 25 Frs >> X-rays 100 Frs	X-rays 25 Frs >> chronic γ-rays	X-rays 100 Frs = chronic γ-rays
	Overall difference judged from all comparisons[d]	X-rays 25 Frs ≥ acute X-rays > X-rays 100 Frs = chronic γ-rays					
6 and 12	Mean score[f]	0.2	0.3	0.6	0.6	0.8	0.3
	Difference judged from each comparison[c]	X-rays 25 Frs ≥ acute X-rays	Acute X-rays > X-rays 100 Frs	Acute X-rays >> chronic γ-rays	X-rays 25 Frs >> X-rays 100 Frs	X-rays 25 Frs >> chronic γ-rays	X-rays 100 Frs > chronic γ-rays
	Overall difference judged from all comparisons[d]	X-rays 25 Frs ≥ acute X-rays > X-rays 100 Frs > chronic γ-rays					

Fr, fractions. IMT, intima-media thickness. IMT-IF, IMT determined from immunofluorescence images. IMT-HC, IMT determined from histochemistry images. [a] Criteria for "A vs B" comparisons: ** = 1.5 (A > B), * = 1 (A > B), # = 0.5 (A > B), ns = 0 (A = B). [b] Mean score ranged from 0.18 (X-rays 25 Frs vs acute X-rays) to 0.93 (X-rays 25 Frs vs chronic γ-rays). [c] Criteria for mean scores: >> (mean score ≥ 0.5), > (0.2 ≤ mean score < 0.5), ≥ (0.1 ≤ mean score < 0.2), = (mean score < 0.1). [d] Judged from differences in each of the 6 comparisons shown immediately above. [e] Mean score ranged from 0.08 (X-rays 100 Frs vs chronic γ-rays) to 0.58 (X-rays 25 Frs vs chronic γ-rays). [f] Mean score ranged from 0.15 (X-rays 25 Frs vs acute X-rays) to 0.75 (X-rays 25 Frs vs chronic γ-rays).

Table S7. Comparison of radiation effects in the aorta at 6 months after starting irradiation, averaged over 20 endpoints among four irradiation regimens.

Alternative hypotheses[a]	One-sided p values for equal averaged effects between the given irradiation conditions A vs B[b]					
	X-rays 25 Frs vs acute X-rays	Acute X-rays vs X-rays 100 Frs	Acute X-rays vs chronic γ-rays	X-rays 25 Frs vs X-rays 100 Frs	X-rays 25 Frs vs chronic γ-rays	X-rays 100 Frs vs chronic γ-rays
$\|A\| > \|B\|$	3×10^{-2}	3×10^{-6}	1×10^{-13}	3×10^{-8}	1×10^{-14}	4×10^{-5}
$\|A\| < \|B\|$	9×10^{-1}	1×10^{0}	1×10^{0}	1×10^{0}	1×10^{0}	1×10^{0}
Difference in each comparison[c]	X-rays 25 Frs > acute X-rays	Acute X-rays >> X-rays 100 Frs	Acute X-rays >> chronic γ-rays	X-rays 25 Frs >> X-rays 100 Frs	X-rays 25 Frs >> chronic γ-rays	X-rays 100 Frs > chronic γ-rays
Overall difference[d]	X-rays 25 Frs > acute X-rays >> X-rays 100 Frs > chronic γ-rays					

Frs, fractions. [a] vs null hypothesis that the absolute values of the radiation effects between the given comparison groups are equal for all endpoints ($|A| = |B|$). [b] p-value of one-sided Kolmogorov–Smirnov goodness-of-fit test for the null hypothesis that the distribution of the p values from the individual tests (in Table S6) is the standard uniform, which is true with no difference in radiation effects for all endpoints between the given comparison groups. [c] Criteria for p values for $|A| > |B|$: >> ($p < 1 \times 10^{-5}$), > ($1 \times 10^{-5} \leq p < 1 \times 10^{-1}$). [d] Judged from differences in each of the 6 comparisons shown immediately above.

Table S8. Comparison of radiation effects in the aorta at 12 months after starting irradiation, averaged over 20 endpoints among four irradiation regimens.

Alternative hypotheses[a]	One-sided p values for equal averaged effects between the given irradiation conditions A vs B[b]					
	X-rays 25 Frs vs acute X-rays	Acute X-rays vs X-rays 100 Frs	Acute X-rays vs chronic γ-rays	X-rays 25 Frs vs X-rays 100 Frs	X-rays 25 Frs vs chronic γ-rays	X-rays 100 Frs vs chronic γ-rays
$\|A\| > \|B\|$	5×10^{-2}	2×10^{-5}	1×10^{-4}	8×10^{-9}	8×10^{-11}	5×10^{-1}
$\|A\| < \|B\|$	8×10^{-1}	1×10^{0}	1×10^{0}	1×10^{0}	1×10^{0}	6×10^{-1}
Difference in each comparison[c]	X-rays 25 Frs > acute X-rays	Acute X-rays > X-rays 100 Frs	Acute X-rays > chronic γ-rays	X-rays 25 Frs >> X-rays 100 Frs	X-rays 25 Frs >> chronic γ-rays	X-rays 100 Frs = chronic γ-rays
Overall difference[d]	X-rays 25 Frs > acute X-rays > X-rays 100 Frs = chronic γ-rays					

Frs, fractions. [a] vs null hypothesis that the absolute values of the radiation effects between the given comparison groups are equal for all endpoints ($|A| = |B|$). [b] p-value of one-sided Kolmogorov–Smirnov goodness-of-fit test for the null hypothesis that the distribution of the p values from the individual tests (in Table S6) is the standard uniform, which is true with no difference in radiation effects for all endpoints between the given comparison groups. [c] Criteria for p values for $|A| > |B$: >> ($p < 1 \times 10^{-5}$), > ($1 \times 10^{-5} \leq p < 1 \times 10^{-1}$), ≥ ($1 \times 10^{-1} \leq p < 3 \times 10^{-1}$), = ($p \geq 3 \times 10^{-5}$). [d] Judged from differences in each of the 6 comparisons shown immediately above.

Table S9. Comparison of radiation effects in the aorta at 6 and 12 months after starting irradiation, averaged over 40 endpoints among four irradiation regimens.

Alternative hypotheses[a]	One-sided p values for equal averaged effects between the given irradiation conditions A vs B[b]					
	X-rays 25 Frs vs acute X-rays	Acute X-rays vs X-rays 100 Frs	Acute X-rays vs chronic γ-rays	X-rays 25 Frs vs X-rays 100 Frs	X-rays 25 Frs vs chronic γ-rays	X-rays 100 Frs vs chronic γ-rays
$\|A\| > \|B\|$	1×10^{-2}	1×10^{-10}	3×10^{-14}	4×10^{-14}	0×10^{0}	1×10^{-3}
$\|A\| < \|B\|$	9×10^{-1}	1×10^{0}	1×10^{0}	1×10^{0}	1×10^{0}	1×10^{0}
Difference in each comparison[c]	X-rays 25 Frs > acute X-rays	Acute X-rays >> X-rays 100 Frs	Acute X-rays >> chronic γ-rays	X-rays 25 Frs >> X-rays 100 Frs	X-rays 25 Frs >> chronic γ-rays	X-rays 100 Frs > chronic γ-rays
Overall difference[d]	X-rays 25 Frs > acute X-rays >> X-rays 100 Frs > chronic γ-rays					

Frs, fractions. [a] vs null hypothesis that the absolute values of the radiation effects between the given comparison groups are equal for all endpoints ($|A| = |B|$). [b] p-value of one-sided Kolmogorov–Smirnov goodness-of-fit test for the null hypothesis that the distribution of the p values from the individual tests (in Table S6) is the standard uniform, which is true with no difference in radiation effects for all endpoints between the given comparison groups. [c] Criteria for p values for $|A| > |B|$: >> ($p < 1 \times 10^{-5}$), > ($1 \times 10^{-5} \leq p < 1 \times 10^{-1}$). [d] Judged from differences in each of the 6 comparisons shown immediately above.

References

1. Hamada, N.; Kawano, K.I.; Nomura, T; Furukawa, K.; Yusoff, F.M.; Maruhashi, T.; Maeda, M.; Nakashima, A.; Higashi, Y. Vascular Damage in the Aorta of Wild-Type Mice exposed to Ionizing Radiation: Sparing and Enhancing Effects of Dose Protraction. *Cancers* **2021**, *13*, 5344, doi:10.3390/cancers13215344.

2. Hamada, N.; Kawano, K.I.; Yusoff, F.M.; Furukawa, K.; Nakashima, A.; Maeda, M.; Yasuda, H.; Maruhashi, T.; Higashi, Y. Ionizing Irradiation Induces Vascular Damage in the Aorta of Wild-Type Mice. *Cancers* **2020**, *12*, 3030, doi:10.3390/cancers12103030.
